# A multimodal multi-agent LLM framework for identifying key drivers of sleep disorders

**DOI:** 10.3389/fneur.2026.1851192

**Published:** 2026-07-06

**Authors:** Chongyang Fu, Syed Kamaruzaman Bin Syed Ali, Mohd Shahril Nizam Bin Shaharom

**Affiliations:** 1 Department of Educational Foundations and Humanities, Faculty of Education, University of Malaya, Kuala Lumpur, Malaysia; 2 Department of Curriculum and Instructional Technology, Faculty of Education, University of Malaya, Kuala Lumpur, Malaysia

**Keywords:** multi-agent large language models, multivariate analysis, sleep disorders, sleep health, sleep quality

## Abstract

**Introduction:**

Sleep quality and sleep disorders are influenced by interacting lifestyle, behavioral, physiological, and occupational determinants, but most existing studies examine these factors in isolation. Traditional statistical methods may be limited in modeling complex interactions, while many machine-learning approaches remain insufficiently interpretable for clinically meaningful sleep research.

**Methods:**

We developed an interpretable large language model (LLM)-based multi-agent multimodal framework for sleep disorder analysis. The framework includes three specialized agents: a Data Analyst Agent for identifying correlations, feature relevance, and interaction effects; a Physiology and Health Analyst Agent for contextual interpretation; and a Validation Analyst Agent for evaluating evidential grounding and consistency. The framework was applied to public and synthetic sleep-health datasets.

**Results:**

Pairwise bootstrap analyses showed weak or uncertain associations between bedtime consistency, light exposure, caffeine intake, stress, heart rate, and continuous sleep-duration or sleep-quality outcomes, whereas high caffeine intake was associated with elevated sleep-disorder risk. Physical activity effects differed by activity type: agility drills, jump tests, and lateral moves were more frequently linked to insomnia, whereas endurance running showed a stronger association with sleep apnea. Occupational context, psychological stress, stimulant use, and physiological indicators jointly influenced sleep disorder profiles, although several pairwise physiological associations remained weak and should be interpreted cautiously.

**Discussion:**

The proposed framework enhances interpretability, supports evidence-grounded reasoning, and reduces unsupported claims in multimodal sleep analysis. Because the second dataset was synthetic, the cross-dataset analysis should be interpreted as a controlled distributional robustness check rather than external clinical validation or evidence of broad clinical generalizability.

## 1 Introduction

Sleep is a fundamental biological process that supports cognitive performance, metabolic homeostasis, cardiovascular regulation, and overall human health (1). In sleep medicine, disturbances in sleep quality, sleep duration, and sleep regulation are increasingly recognized not only as contributors to impaired daily functioning and reduced quality of life, but also as factors associated with brain dysfunction, cognitive impairment, and broader neurological consequences (2). Sleep disorders such as insomnia, sleep apnea, and circadian disruption are now understood as multifactorial conditions shaped by interacting behavioral, physiological, environmental, and occupational influences. Consequently, improving the analytical understanding of sleep-related determinants has become an important goal for sleep medicine research, particularly in the context of developing more precise, interpretable, and clinically meaningful computational frameworks (3).

Despite growing interest in the determinants of sleep health, existing analytical approaches often remain fragmented. Many studies examine isolated variables such as caffeine intake, bedtime regularity, physical activity, psychological stress, or physiological status, but do not adequately characterize how these heterogeneous factors interact to jointly influence sleep quality and sleep disorder risk (4). Traditional statistical approaches are often limited by linear assumptions, predefined hypotheses, and restricted capacity to model higher-order dependencies across multiple domains (5). Likewise, conventional machine learning and deep learning models can identify associations or produce predictions, yet frequently operate as black boxes with limited interpretability, thereby restricting their translational value for sleep medicine applications (6). This methodological gap is particularly important in medically relevant sleep analysis, where transparent reasoning, domain grounding, and integrated interpretation of heterogeneous variables are essential.

Recent advances in artificial intelligence, especially large language models (LLMs), have created new opportunities for more interpretable and integrative analysis of complex medical data (7). In contrast to conventional predictive models, LLM-based systems can support structured reasoning over heterogeneous evidence, synthesize patterns across multiple variable types, and produce domain-aware explanations. When organized as multi-agent frameworks, LLMs can emulate complementary expert roles, iteratively validate analytical claims, and provide more transparent reasoning processes for multivariate decision-making (8, 9). In treatment management contexts, re-thinking the nature of planning has enabled safe and personalized therapeutic strategies through LLM-based coordination (10). This demonstrates that agent specialization—mirroring our Data Analyst, Physiology, and Validation agents—can improve both safety and personalization in clinical AI systems. These properties make them especially attractive for sleep medicine research, where clinically meaningful insight often depends on understanding interactions among behavioral, physiological, occupational, and lifestyle determinants rather than treating them as isolated predictors. From this perspective, LLM-based multi-agent systems can be viewed as a promising direction for multimodal sleep-health analysis, even when the underlying data originate from heterogeneous tabular and clinical domains rather than a single unimodal source.

Several gaps in the current literature motivate the present study. First, although prior studies have reported associations between lifestyle-related factors such as bedtime consistency, light exposure, and caffeine consumption and sleep outcomes, these investigations commonly evaluate such variables independently or through limited pairwise analysis (11, 12). As a result, the joint and potentially synergistic effects of multiple behavioral factors on sleep duration and sleep quality remain insufficiently characterized (5, 13–16). Yet, from a sleep medicine perspective, these factors are unlikely to act in isolation; instead, they interact within broader behavioral and physiological contexts that require integrated analysis.

Second, the relationship between physical activity and sleep disorders remains inconsistently reported, partly because prior work often fails to distinguish the effects of activity type, intensity, and duration (17, 18). Evidence suggests that different forms of physical activity may have distinct associations with insomnia, sleep apnea, and related sleep outcomes, but many existing studies treat physical activity as a homogeneous predictor (19–23). This limits understanding of how activity-related exposures interact with sleep duration, sleep quality, and individual physiological characteristics. More nuanced modeling is therefore needed to identify interpretable patterns that may be useful for sleep-health stratification and future intervention-oriented research.

Third, the combined impact of caffeine intake, psychological stress, and physiological factors across occupational groups remains underexplored (24, 25). Existing studies often consider these determinants separately, despite the likelihood that they exert synergistic or antagonistic effects on sleep disorder risk. For example, both high caffeine intake and elevated stress have been associated with insomnia-related outcomes, yet their combined influence may differ substantially depending on occupational context, physiological state, and broader behavioral conditions (26, 27). Furthermore, physiological indicators such as heart rate and body temperature are rarely integrated with behavioral and occupational variables within a single interpretable analytical framework (28–30). This separation limits the field's ability to move toward more comprehensive, medically meaningful analysis of sleep disorders.

Beyond these domain-specific gaps, there remains a broader methodological limitation. Traditional statistical, machine learning, and deep learning approaches are generally constrained either by limited interaction modeling, insufficient interpretability, or dependence on pre-specified analytical structures (31, 32). Such methods also make limited use of contextual knowledge relevant to sleep medicine and are not designed to perform structured, evidence-aware reasoning across heterogeneous determinant categories. In contrast, LLM-based multi-agent systems offer a new computational paradigm in which analytical extraction, domain interpretation, and iterative validation can be explicitly separated into collaborative reasoning components. This makes them particularly suitable for medical data integration problems where transparency and interpretability are critical.

**Figure 1:**
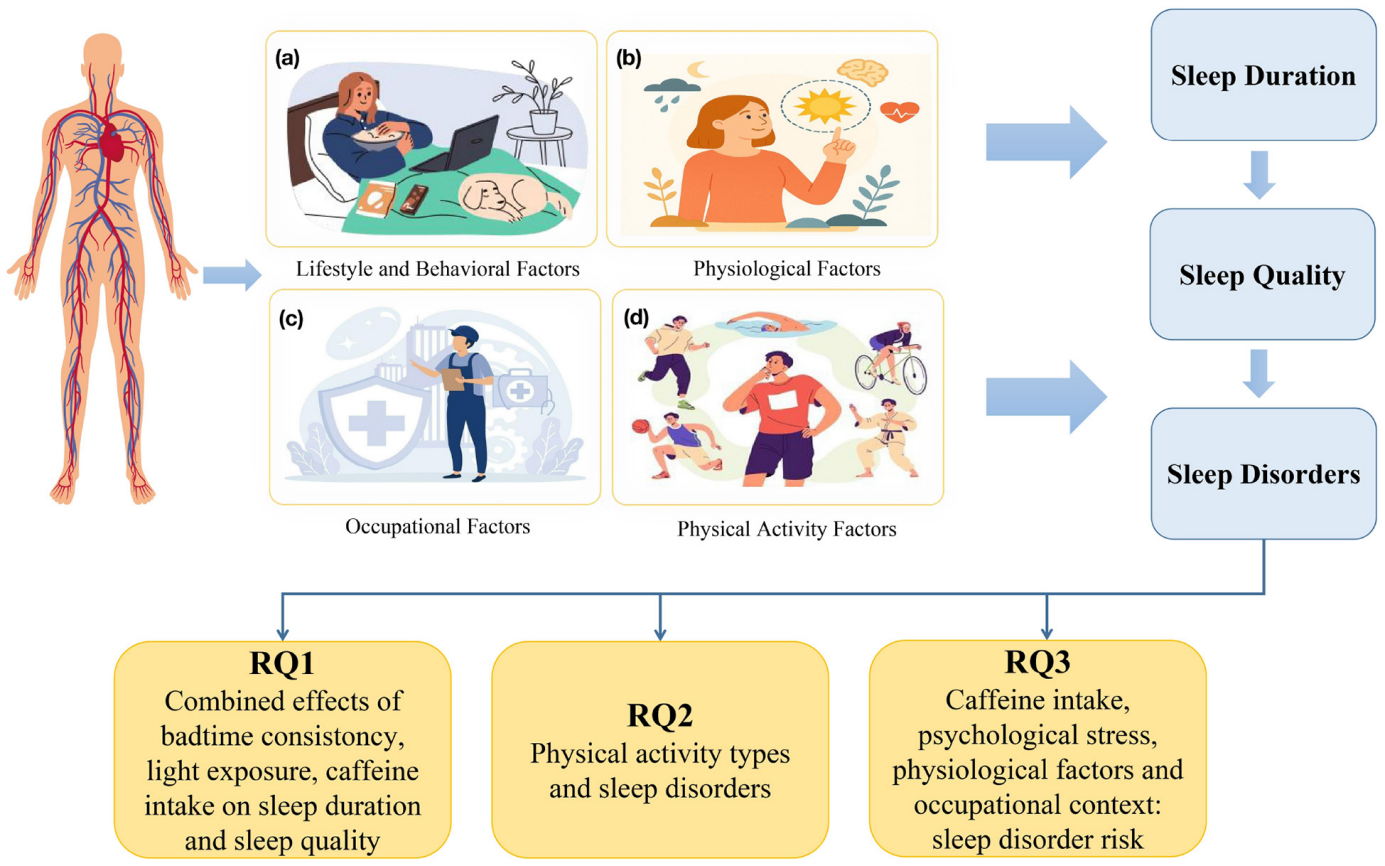
Process of research question formulation. The framework integrates four input domains: **(a)** lifestyle and behavioral factors, **(b)** physiological factors, **(c)** occupational factors, and **(d)** physical activity factors. These domains interact to influence sleep duration, sleep quality, and sleep disorders. Research questions (RQ1–RQ3) are derived to systematically examine combined behavioral effects, the role of physical activity, and the interplay of caffeine, stress, and physiology across occupations.

Motivated by these needs, this study develops an interpretable LLM-based multi-agent multivariate framework for multimodal analysis of sleep quality and sleep disorders. The proposed framework organizes heterogeneous information from four complementary domains—lifestyle and behavioral factors, physiological factors, occupational context, and physical activity—and maps these domains to sleep duration, sleep quality, and sleep-disorder outcomes. As illustrated in Figure 1, these domains provide the basis for three targeted research questions (RQ1–RQ3). The first examines the combined influence of bedtime consistency, light exposure, and caffeine intake on sleep outcomes. The second investigates how physical activity type and intensity relate to the prevalence of sleep disorders. The third evaluates how caffeine intake, psychological stress, and physiological factors interact across occupational groups to shape sleep health.

Unlike conventional approaches that either analyze isolated variables or provide opaque predictions, the proposed framework combines multivariate evidence extraction with domain-aware multi-agent reasoning and iterative validation. In this way, it is intended to support transparent identification of clinically relevant interaction patterns across heterogeneous determinants, thereby advancing interpretable AI for sleep medicine research. Although the present study does not directly model neurodegenerative disease trajectories, it is motivated by the broader recognition that sleep disorders are closely connected to brain dysfunction and cognitive impairment, making improved analytical understanding of sleep-related determinants relevant to the wider study of neurological health.

The objectives of this study are threefold:

1. to quantify the associations of bedtime consistency, light exposure, and caffeine intake with sleep duration and sleep quality;
2. to examine how different physical activity types relate to the prevalence and profile of sleep disorders;
3. to assess how caffeine intake, psychological stress, physiological factors, and occupational context interact to modulate sleep disorder risk.

## 2 Method

### 2.1 Overview of the proposed framework

**Figure 2:**
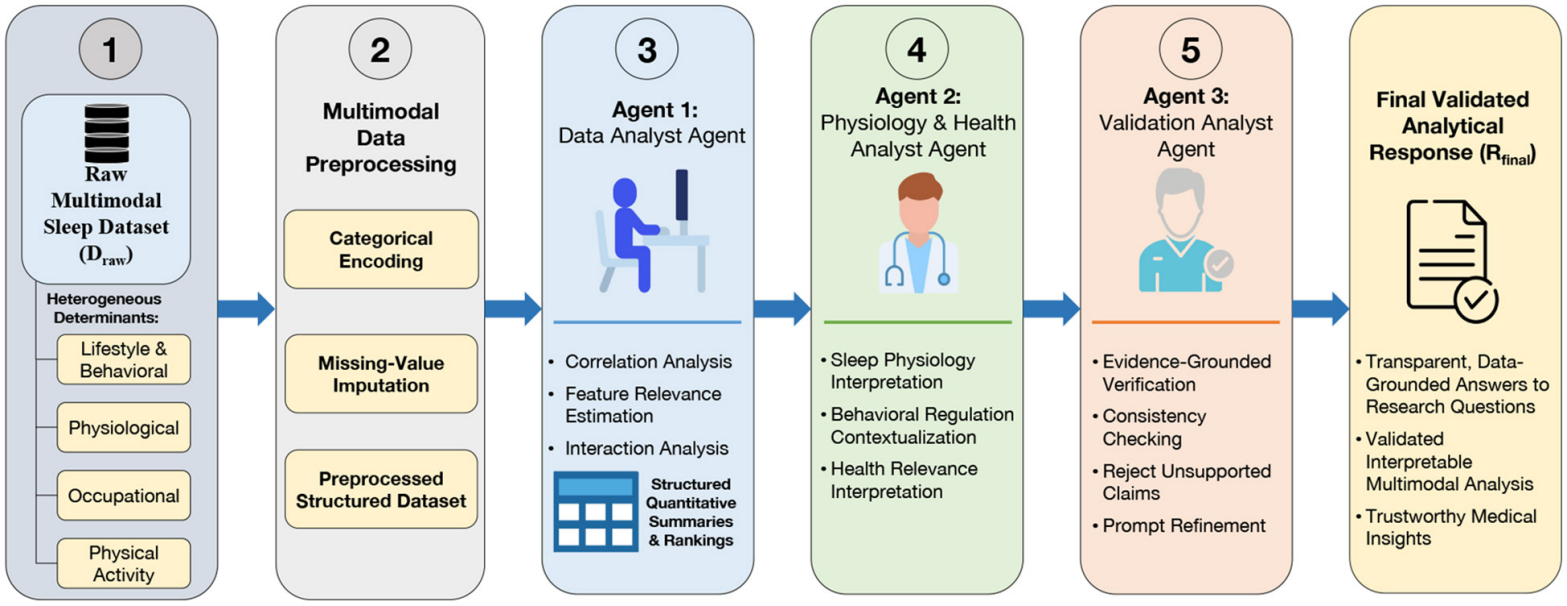
Static pipeline overview of the multi-agent analytical framework.

**Figure 3:**
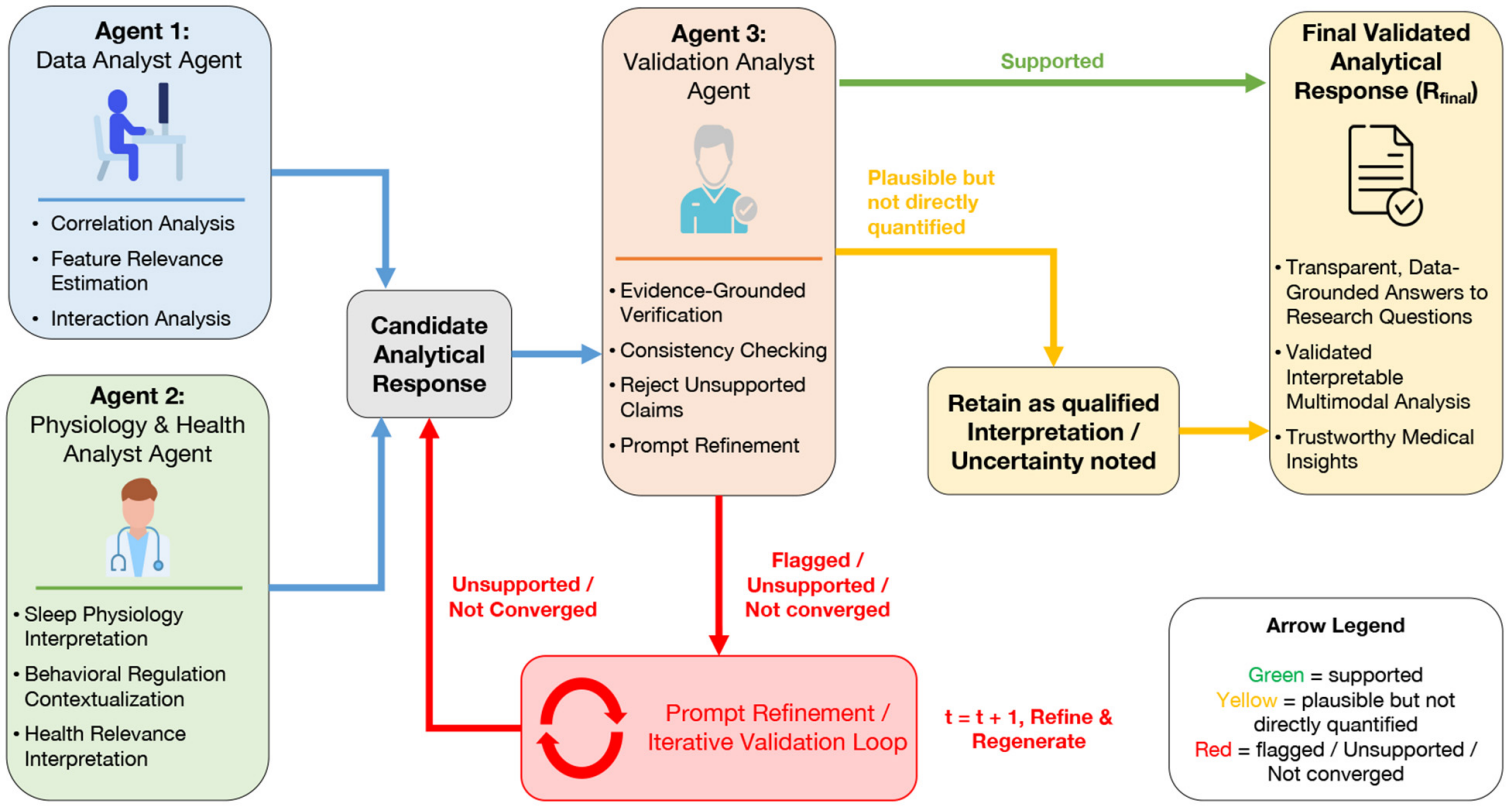
Validation loop and claim-validation logic of the multi-agent analytical framework.

To support interpretable multimodal analysis in sleep medicine, we developed a large language model (LLM)-based multi-agent framework for investigating heterogeneous determinants of sleep quality and sleep disorders. In the context of this study, *multimodal* refers to the integration of complementary sleep-relevant information from multiple domains, including lifestyle and behavioral variables, physiological measurements, occupational context, and physical activity characteristics. End-to-end transformer architectures with novel ensemble learning methods have demonstrated that integrating imaging data with clinical narratives significantly improves diagnostic performance for complex neurological conditions such as brain stroke (33). This motivates our integration strategy of combining structured tabular determinants rather than treating each domain in isolation. Rather than analyzing these domains independently, the proposed framework treats them as interacting sources of evidence that jointly shape sleep-related outcomes. The complete overview of the proposed method is shown in Figures 2, 3. Figure 2 provides a static left-to-right pipeline overview with five numbered steps for traceability, whereas Figure 3 zooms into the iterative validation loop and explicitly illustrates the color-coded claim-validation logic.

The system consists of three specialized agents: (i) a *Data Analyst Agent* for extracting statistical relationships, feature relevance, and multivariate interaction effects from structured data; (ii) a *Physiology and Health Analyst Agent* for contextualizing these patterns in relation to sleep physiology, behavioral regulation, and health relevance; and (iii) a *Validation Analyst Agent* for evidence-grounded verification, consistency checking, and iterative refinement. Through this collaborative design, the framework aims to generate transparent, domain-informed, and data-grounded analytical insights that extend beyond conventional statistical analysis and opaque machine learning prediction.

To clarify the role of each agent in producing the final validated response, Table 1 summarizes the agent-specific inputs, analytical operations, intermediate outputs, and contributions to the final evidence-grounded interpretation.

**Table 1:** Agent-specific contributions to the final validated responses.

Agent	Input	Main analytical operation	Intermediate output	Contribution to final validated response
Data Analyst Agent	Preprocessed structured dataset; research question-specific variables; sleep-related outcomes	Computes correlations, feature relevance, pairwise interaction effects, subgroup summaries, and ranked determinant lists	Structured quantitative evidence summary, including effect sizes, ranked features, interaction patterns, and null or weak findings	Provides the numerical evidence base for the final response and prevents unsupported interpretation by requiring all claims to originate from dataset-derived statistics
Physiology and Health Analyst Agent	Structured quantitative evidence generated by the Data Analyst Agent	Maps statistical patterns to sleep physiology, behavioral regulation, physical activity context, and health relevance; distinguishes supported, plausible, and speculative interpretations	Domain-grounded explanatory narrative linking statistical results to sleep medicine concepts	Transforms numerical findings into clinically meaningful interpretation while avoiding claims that are not directly or plausibly supported by the extracted evidence
Validation Analyst Agent	Initial explanatory response; structured quantitative evidence; claim-level evidence links	Checks whether each claim is traceable to dataset-derived evidence; flags unsupported, overgeneralized, or speculative statements; requests revision or removal when necessary	Final validated response with unsupported claims removed or revised and retained claims linked to evidence	Improves trustworthiness by ensuring that the final response is evidence-grounded, internally consistent, and not solely generated from LLM prior knowledge

The three agents operate sequentially and iteratively. The Data Analyst Agent generates the quantitative evidence base, the Physiology and Health Analyst Agent provides domain interpretation, and the Validation Analyst Agent verifies claim-level support before producing the final validated response.

As shown in Table 1, the final validated response is not produced by a single unconstrained LLM output. Instead, it is assembled through a staged process in which quantitative evidence is first extracted, then interpreted in a sleep medicine context, and finally verified against the dataset-derived evidence. This design allows the framework to separate statistical extraction, domain interpretation, and evidence validation, thereby improving transparency and reducing the risk of unsupported or hallucinated claims.

### 2.2 Problem formulation

Let the preprocessed dataset be denoted as Equation 1.


$$\begin{array}{l} D=\left\{\left(x_{i},y_{i}\right)|i=1,2,\ldots ,N\right\}, \end{array}$$ (1)

where $x_{i}\in {\mathbb{R}}^{d}$ is the *d*-dimensional feature vector for subject *i*, containing heterogeneous determinants spanning behavioral, physiological, occupational, and physical-activity domains, and *y*_*i*_ denotes the corresponding sleep-related outcome. Depending on the research question, *y*_*i*_ may represent sleep duration, sleep quality, or sleep disorder status.

The goal of the framework is not restricted to predicting *y*_*i*_. Instead, the objective is to identify interpretable multivariate patterns within heterogeneous sleep-related data, generate domain-grounded explanations for those patterns, and validate whether the resulting claims are supported by dataset-derived evidence. The method is therefore designed as an interpretable multimodal analytical framework for sleep medicine rather than a purely predictive black-box model.

### 2.3 Data representation and preprocessing

Preprocessing provides the foundation for consistent multimodal analysis of heterogeneous sleep-related determinants. Because the input data include variables from multiple domains with different scales and formats, two primary preprocessing operations were performed: categorical encoding and missing-value imputation. Similar ensemble-based preprocessing strategies have proven effective in respiratory disease detection, where multi-layer ensemble deep learning frameworks combined with feature ranking methods achieved robust COVID-19 identification from cough audio signals (34). This supports our staged approach of feature extraction followed by domain-specific analysis.

#### 2.3.1 Encoding of categorical variables

Categorical variables, such as occupation and BMI category, were transformed using one-hot encoding in order to avoid introducing artificial ordinal structure. Formally, a categorical attribute *x*_*ij*_ is encoded as Equation 2.


$$\begin{array}{l} \text{Enc}\left(x_{ij}\right)={\text{e}}_{k},\;{\text{e}}_{k}\in {\left\{0,1\right\}}^{K}, \end{array}$$ (2)

where **e**_*k*_ is a binary vector of length *K* with one activated entry corresponding to category *k*.

#### 2.3.2 Imputation of missing values

To reduce sample loss and maintain analytical consistency, missing values were imputed prior to downstream analysis. For continuous variables, median imputation was used; for categorical variables, mode imputation was applied. For a continuous feature *x*_*ij*_, the imputed value is defined as Equation 3.


$$\begin{array}{l} x_{ij}^{\prime }=\{\begin{array}{ll} x_{ij}, & \text{if }x_{ij}\neq \emptyset ,\\ \text{median}\left(x_{j}\right), & \text{otherwise}. \end{array} \end{array}$$ (3)

These preprocessing steps produce a unified tabular representation suitable for structured multivariate analysis and subsequent LLM-based agentic reasoning.

### 2.4 Comparison with existing interpretable approaches

To contextualize the methodological contribution without overstating novelty, we compared the proposed framework with conventional interpretable and explainable approaches used in sleep medicine. Penalized logistic regression, XGBoost, SHAP-based explanation, and Bayesian network models have all been productively applied to sleep-disorder prediction or risk modeling (5, 28, 35–38). These approaches should therefore be understood as substantive baselines rather than as straw-man alternatives.

Bayesian networks can incorporate expert-defined structures or data-driven structure learning and provide probabilistic interpretability. Their limitation in the present context is not a lack of structure learning, but the need to select and validate graph-learning assumptions and the absence of built-in claim-level textual validation. Similarly, SHAP is a model-agnostic post-hoc explanation framework that provides local and global feature attributions. Its limitation in the present context is that attribution scores alone do not generate sleep-physiology interpretation or verify whether narrative clinical claims are traceable to dataset-derived evidence.

The proposed framework is therefore not claimed to be a universally superior predictive classifier. Its intended contribution is to combine deterministic statistical evidence extraction, sleep-medicine interpretation, and explicit validation of textual claims. The quantitative benchmark below was added to assess whether the variables retained by the agentic framework preserve useful predictive signal relative to standard supervised baselines.

### 2.5 Predictive benchmark against conventional baselines

To address whether the proposed framework preserves predictive signal relative to conventional approaches, we conducted a supervised benchmark on the same preprocessed benchmark dataset. The benchmark was not intended to redefine the proposed framework as a purely predictive classifier, but to contextualize the predictive utility of the evidence-validated predictive pipeline identified by the multi-agent system. We compared three models: penalized logistic regression, XGBoost with SHAP-based interpretation, and the proposed multi-agent validated predictive framework using a class-balanced ExtraTrees supervised head. All models used the same target definition and preprocessing pipeline, including median imputation for continuous variables, mode imputation for categorical variables, one-hot encoding for categorical predictors, and stratified 5-fold cross-validation. The synthetic distributional robustness dataset was not used to train, tune, or rank the Table 2 benchmark models. Performance was evaluated using accuracy, macro-F1, weighted-F1, and one-vs.-rest macro-AUC.

**Table 2:** Predictive benchmark against conventional supervised baselines.

Model	Feature set	Accuracy	Macro-F1	Weighted-F1	Macro-AUC
Penalized logistic regression	All preprocessed predictors	0.764	0.410	0.793	0.707
XGBoost + SHAP	All preprocessed predictors	0.823	0.430	0.817	0.655
Proposed multi-agent validated feature framework	Agent-validated predictive feature set	0.921	0.609	0.889	0.758

As shown in Table 2, the proposed multi-agent validated feature framework achieved the strongest overall benchmark performance among the three evaluated models, with an accuracy of 0.921, macro-F1 of 0.609, weighted-F1 of 0.889, and macro-AUC of 0.758. The improvement was most pronounced for accuracy and weighted-F1, whereas macro-F1 remained lower because the minority sleep-disorder subtypes were sparsely represented. These results indicate that the agent-validated predictive instantiation retained useful class-discriminative signal while preserving the framework's primary emphasis on interpretable evidence extraction and claim validation.

### 2.6 Multi-agent LLM framework for multimodal sleep analysis

Following preprocessing, the structured dataset was analyzed using a multi-agent LLM system designed for interpretable reasoning over heterogeneous sleep-related determinants. The framework combines quantitative evidence extraction, domain-aware interpretation, and iterative validation in a unified analytical workflow. The unified analytical workflow is formalized in Equation 4:


$$\begin{array}{l} R_{\text{final}}=V\left(H\left(D,A\left(D\right)\right)\right), \end{array}$$ (4)

where A denotes the Data Analyst Agent, H denotes the Physiology and Health Analyst Agent, and V denotes the Validation Analyst Agent.

In this formulation, A first extracts structured multivariate evidence from the dataset, H translates the extracted patterns into sleep medicine interpretations, and V verifies whether these interpretations are supported by the underlying data.

### 2.7 LLM implementation and reproducibility details

All LLM-based repeated-generation analyses were conducted using llama-3.1-8b-instant via the Groq API. Unless otherwise stated, the generation parameters were: temperature = 0.2, top-*p* = 1.0, maximum output tokens = 1,000, and no frequency or presence penalty. The Groq API does not provide a fixed-seed guarantee for this workflow; therefore, repeated-run stability was evaluated through *N* = 5 independent repeated generations.

The Data Analyst Agent relied on deterministic statistical scripts for correlations, feature relevance estimation, interaction screening, bootstrap confidence intervals, and predictive benchmarking. The LLM agents received structured numerical summaries rather than raw participant-level identifiers. The Physiology and Health Analyst Agent generated domain-grounded interpretations based on these summaries, and the Validation Analyst Agent checked whether each retained claim could be traced to dataset-derived evidence.

### 2.8 Data analyst agent

The Data Analyst Agent was designed to extract dataset-grounded quantitative patterns linking heterogeneous determinants to sleep-related outcomes. Its role is to produce structured statistical evidence that can subsequently be interpreted in a sleep medicine context. Specifically, the agent performs three analytical operations: correlation analysis, feature relevance estimation, and interaction analysis.

#### 2.8.1 Correlation analysis

To characterize pairwise associations between predictors and sleep outcomes, correlation coefficients were computed as Equation 5.


$$\begin{array}{l} \rho_{j,y}=\frac{\text{Cov}\left(x_{j},y\right)}{\sigma_{x_{j}}\sigma_{y}}, \end{array}$$ (5)

where ρ_*j,y*_ denotes the correlation between feature *x*_*j*_ and outcome *y*, Cov(·) denotes covariance, and σ denotes standard deviation.

#### 2.8.2 Feature relevance estimation

To quantify the relative influence of individual predictors on sleep-related outcomes, feature relevance was estimated using model-derived importance scores computed from the structured analytical pipeline. Formally, the relevance of feature *x*_*j*_ is denoted as Equation 6.


$$\begin{array}{l} I_{j}=\text{Imp}\left(x_{j}\right), \end{array}$$ (6)

where Imp(·) denotes the importance assigned to feature *x*_*j*_ based on its contribution to the corresponding sleep-related outcome.

#### 2.8.3 Interaction analysis

Because sleep quality and sleep disorders are often shaped by interacting factors rather than isolated variables, pairwise interaction effects were also examined, as formalized in Equation 7:


$$\begin{array}{l} \Delta_{j,k}=\text{Int}\left(x_{j},x_{k}\right), \end{array}$$ (7)

where Δ_*j,k*_ captures the interaction effect between features *x*_*j*_ and *x*_*k*_ within the analytical pipeline.

The complete procedure of the Data Analyst Agent is formalized in Algorithm 1.

Algorithm 1 **Data analyst agent.**

**Require**:  Preprocessed dataset 𝒟, research question RQ, permutation-defined interaction threshold δ
**Ensure**:  Structured quantitative summary *S*_data_
1:  **for** each feature *x*_*j*_ ∈ 𝒟 **do**
2:    Compute correlation ρ_*j,y*_ with outcome *y* using Eq. (5)
3:    Estimate feature relevance *I*_*j*_ using Eq. (6)
4:  **end for**
5:  **for** each feature pair (*x*_*j*_, *x*_*k*_) where *j* ≠ *k* **do**
6:    Compute pairwise interaction Δ_*j,k*_ using Eq. (7)
7:    **if** |Δ_*j,k*_| > δ **then**
8:    Retain (*x*_*j*_, *x*_*k*_) as a candidate interaction pair
9:    **end if**
10:  **end for**
11:  Rank features by |*I*_*j*_| in descending order
12:  Perform subgroup stratification by occupation, activity type, and disorder category
13:  Compile *S*_data_ ← {ρ_*j,y*_, *I*_*j*_, Δ_*j,k*_,
 subgroup summaries, ranked feature list}
14:  Verify: flag any element of *S*_data_ lacking direct numerical support; remove or mark as inconclusive
15:  **return** *S*_data_

The interaction threshold δ in Algorithm 1 was set using a permutation-based null distribution rather than chosen arbitrarily. Specifically, the sleep-related outcome was permuted *B*_perm_ times, and pairwise interaction effects were recomputed under each permutation. The threshold was formalized in Equation 8:


$$\begin{array}{l} \delta =Q_{0.95}\left(\left\{|\Delta_{j,k}^{*\left(b\right)}|:b=1,\ldots ,B_{\text{perm}},j<k\right\}\right), \end{array}$$ (8)

where $\Delta_{j,k}^{*\left(b\right)}$ denotes the interaction effect estimated under permutation *b*, and *Q*_0.95_ denotes the 95th percentile of the empirical null distribution. Only feature pairs with |Δ_*j,k*_| > δ were retained as candidate interaction pairs. This data-adaptive threshold was used to reduce retention of trivial or noise-driven interaction effects while preserving interpretable pairwise patterns for subsequent physiological interpretation.

The outputs of the Data Analyst Agent were represented as structured numerical summaries, tables, and variable rankings. These structured outputs were then provided to the Physiology and Health Analyst Agent.

#### 2.8.4 Covariate-adjusted analysis of occupational heterogeneity

To examine occupational heterogeneity in sleep disorder subtype and evaluate whether these patterns changed after controlling for demographic and anthropometric covariates, we conducted additional covariate-adjusted analyses. The dependent variable was sleep disorder subtype, coded as insomnia vs. sleep apnea.

Occupation was treated as the focal categorical predictor, while age, gender, and BMI category were included as covariates. The adjusted model was formalized in Equation 9:


$$\begin{array}{r} \log\left(\frac{P\left(Y_{i}=\text{Insomnia}\right)}{P\left(Y_{i}=\text{Sleep Apnea}\right)}\right)=\beta_{0}+\beta_{1}{\text{Occupation}}_{i}+\beta_{2}{\text{Age}}_{i}\\ +\beta_{3}{\text{Gender}}_{i}+\beta_{4}{\text{BMI}}_{i}, \end{array}$$ (9)

where *Y*_*i*_ denotes the sleep disorder subtype for participant *i*. Separate adjusted models were fitted for the public sleep-health dataset and the synthetic distributional robustness dataset. Because several occupation-by-disorder subtype cells were sparse or near-completely separated in the descriptive plots, penalized logistic regression was used to improve estimation stability. Complete or near-complete separation was flagged when an occupation-by-disorder subtype cell contained zero observations or when the unadjusted insomnia proportion was ≤ 5% or ≥95%. Adjusted odds ratios, 95% confidence intervals, adjusted marginal probabilities, and separation status were reported to evaluate whether the observed occupational patterns remained directionally consistent after controlling for age, gender, and BMI category.

#### 2.8.5 Higher-order interactions and scalability

In the present implementation, Equation 7 formally represents pairwise interaction analysis. We focused on pairwise interactions because they provide a transparent and interpretable basis for identifying relationships among heterogeneous sleep-related determinants. Exhaustive estimation of three-way or higher-order interactions was not conducted in the current empirical analysis, because the number of possible combinations increases rapidly as the number of features grows. For a retained feature set of size *K*, the number of pairwise combinations is $\left(\begin{array}{c} K\\ 2 \end{array}\right)$, whereas the number of *h*-order combinations is $\left(\begin{array}{c} K\\ h \end{array}\right)$. As *K* and *h* increase, exhaustive high-order interaction search may substantially increase computational burden, reduce interpretability, and increase the risk of unstable findings, particularly when subgroup sample sizes are limited.

Therefore, higher-order patterns were considered in a constrained and interpretive manner rather than through exhaustive formal interaction testing. Specifically, the framework first identified relevant individual features and pairwise associations. The Physiology and Health Analyst Agent then synthesized supported patterns across behavioral, physiological, occupational, and physical-activity domains. For example, combinations involving caffeine intake, stress level, body temperature, and occupational context were interpreted as joint multi-domain profiles only when the relevant lower-order evidence was available. These patterns were not described as formally estimated higher-order interaction terms unless they were directly supported by explicit interaction analysis.

To improve scalability with increasing feature dimensions, the framework adopts a staged screening strategy. First, features are ranked according to relevance and association strength. Second, pairwise interactions are examined among retained candidate features rather than across all possible variables. Third, possible higher-order combinations are considered only when they satisfy three conditions: the lower-order associations are supported by the data, the combination has a clear sleep-medicine rationale, and the subgroup evidence is sufficiently supported for interpretation. Finally, the Validation Analyst Agent checks whether any higher-order interpretation is supported by dataset-derived evidence. Unsupported, overgeneralized, or unstable higher-order claims are removed, downgraded to inconclusive statements, or rewritten using cautious language.

This design allows the framework to remain interpretable and computationally manageable while still supporting multi-domain reasoning. For larger high-dimensional datasets, the framework can be extended by incorporating feature grouping, domain-based preselection, sparsity constraints, or regularized interaction-selection methods before LLM-based physiological interpretation and validation. Thus, the current study should be understood as emphasizing interpretable pairwise interaction analysis and constrained multi-domain profile interpretation rather than exhaustive high-order interaction modeling.

### 2.9 Physiology and health analyst agent

The Physiology and Health Analyst Agent interprets the quantitative patterns extracted by the Data Analyst Agent in relation to sleep physiology, behavioral regulation, and health relevance. Its purpose is not to generate new statistical evidence, but rather to translate dataset-derived patterns into medically meaningful explanations relevant to sleep medicine. To represent combined behavioral determinants affecting sleep-related outcomes, the framework considers a multivariate relationship of the form shown in Equation 10:


$$\begin{array}{l} {\hat{Y}}_{\text{sleep},i}=\alpha_{0}+\alpha_{1}B_{i}+\alpha_{2}L_{i}+\alpha_{3}C_{i}, \end{array}$$ (10)

where ${\hat{Y}}_{\text{sleep},i}$ may correspond to sleep duration or sleep quality for participant *i*; *B*_*i*_, *L*_*i*_, and *C*_*i*_ denote bedtime consistency, light exposure, and caffeine intake, respectively.

To characterize activity-related effects, we formalize the activity-related contribution in Equation 11:


$$\begin{array}{l} \Delta_{\text{sleep outcome}}^{\text{activity}}=f\left(\text{Intensity},\text{Duration},\text{Type}\right), \end{array}$$ (11)

where *f*(·) represents the contribution of physical activity characteristics to a sleep-related outcome.

For sleep disorder analysis, the probability of disorder risk is formulated as Equation 12.


$$\begin{array}{l} P(\text{Disorder}=1)=\sigma (\beta_{0}+\beta_{1}\text{ Stress}+\beta_{2}\text{ Caffeine}\\ \;+\beta_{3}\text{ Occupation}+\beta_{4}(\text{Stress}\times \text{Caffeine})), \end{array}$$ (12)

where σ(·) is the logistic sigmoid function and β denotes the contribution of stimulant-related, behavioral, and occupational determinants to sleep disorder probability.

The step-by-step procedure of the Physiology and Health Analyst Agent, including the labelling of explanations by evidential support level, is formalized in Algorithm 2.

Algorithm 2 **Physiology and health analyst agent.**

**Require**:  Structured quantitative summary *S*_data_, research question RQ
**Ensure**:  Domain-grounded interpretation report *R*_0_
1:  Parse *S*_data_ to extract top-ranked features, significant correlations, and retained interaction pairs
2:  **for** each retained feature or interaction in *S*_data_ **do**
3:    Map statistical pattern to sleep medicine concept
4:    Examples: bedtime consistency → circadian entrainment; caffeine → adenosine antagonism; heart rate → autonomic arousal
5:    Generate candidate explanation *E*_*k*_ grounded in Eqs. (10)–(12)
6:    Assign support label to *E*_*k*_:
7:    **supported** if directly traceable to *S*_data_
8:    **plausible** if consistent with *S*_data_ but not directly quantified
9:    **speculative** if drawn from prior knowledge only
10:  **end for**
11:  Suppress or clearly flag all **speculative** explanations
12:  Integrate **supported** and **plausible** explanations into a coherent domain narrative per RQ
13:  Assemble *R*_0_ ←
 {*E*_*k*_, evidence links, labels, narrative summary}
14:  **return** *R*_0_

The outputs of this agent consisted of explanatory summaries that link quantitative evidence to physiologically meaningful patterns in sleep medicine.

### 2.10 Validation analyst agent

The Validation Analyst Agent was introduced to address a central challenge in LLM-based medical data analysis: ensuring that generated interpretations remain grounded in the observed dataset rather than reflecting unsupported model priors or memorized background knowledge. This agent verifies whether the claims produced by the previous agents are consistent with the extracted statistics and rejects interpretations that cannot be traced back to dataset-derived evidence.

Each generated claim set *R* was evaluated for evidential support using the binary support function defined in Equation 13:


$$\begin{array}{l} \phi \left(R\right)=\{\begin{array}{ll} 1, & \text{if }R\text{ is supported by the dataset-derived evidence},\\ 0, & \text{otherwise}. \end{array} \end{array}$$ (13)

If a response failed validation, prompt refinement was performed through a bounded, claim-level feedback procedure rather than through open-ended regeneration. Specifically, the Validation Analyst Agent first decomposed the candidate response *R*_*t*_ into a set of individual claims $C\left(R_{t}\right)=\left\{c_{1},c_{2},\ldots ,c_{m}\right\}$. Each claim was then checked against the structured evidence summary *S*_data_ generated by the Data Analyst Agent, including correlation coefficients, effect-size estimates, confidence intervals, subgroup summaries, and interaction results. Unsupported, overgeneralized, or speculative claims were collected into a flagged claim set *F*_*t*_, and the missing evidence required to support these claims was recorded as *M*_*t*_.

To operationalize the convergence threshold ϵ, response stability was evaluated at the level of atomic claims rather than raw text strings. Each claim was represented by five elements: determinant, sleep-related outcome, direction of association, evidential status, and linked evidence source. Two claims from consecutive iterations were considered matched only when they referred to the same determinant and outcome, had the same direction of association, and carried the same evidential status. The claim-level distance between two consecutive responses was defined as Equation 14:


$$\begin{array}{l} d_{\text{claim}}\left(R_{t},R_{t-1}\right)=1-\frac{2|M_{t}|}{\left|C\left(R_{t}\right)|+|C\left(R_{t-1}\right)\right|}, \end{array}$$ (14)

where $M_{t}$ is the set of matched claims between *R*_*t*_ and *R*_*t*−1_. In this study, ϵ was set to 0.10. Thus, the response was considered stable when *d*_claim_(*R*_*t*_, *R*_*t*−1_) < 0.10 and all retained claims were supported by dataset-derived evidence. Purely grammatical or stylistic changes were not counted as substantive claim changes, whereas changes in determinant, outcome, direction, evidence label, or support status were counted as substantive.

The validation-guided prompt refinement signal was therefore defined as a structured feedback message containing the flagged claims, missing evidence requirements, and revision instructions. The regenerated response was produced according to Equation 15:


$$\begin{array}{l} R_{t+1}=f_{\theta }\left(p⊕\Delta p_{t},S_{\text{data}},R_{t}\right),\;\Delta p_{t}=G\left(F_{t},M_{t}\right), \end{array}$$ (15)

where *R*_*t*_ is the response at iteration *t*, *p* is the base prompt, ⊕ denotes appending validation feedback to the base prompt, Δ*p*_*t*_ is the validation-guided refinement signal, G(·) is the feedback construction function, *S*_data_ is the structured dataset-derived evidence, and *f*_θ_ is the underlying LLM generation function. In this formulation, Δ*p*_*t*_ was not an arbitrary prompt modification; it explicitly instructed the model to revise, qualify, or remove claims that could not be traced to the extracted evidence.

The iterative procedure terminated when the response satisfied both the evidence-support and claim-stability criteria, or when the maximum number of refinement iterations was reached. This process is formalized in Equation 16:


$$\begin{array}{r} \left[\frac{1}{\left|C\left(R_{t+1}\right)\right|}\underset{c_{i}\in C\left(R_{t+1}\right)}{\sum }\phi \left(c_{i}\right)=1\right]\wedge \left[d_{\text{claim}}\left(R_{t+1},R_{t}\right)<\epsilon \right],\\ \;t\leq T_{\max}. \end{array}$$ (16)

In practical implementation, the convergence criterion was operationalized at the claim level. The first condition required that all retained claims in the regenerated response were supported by dataset-derived evidence. The second condition required that fewer than 10% of retained claims changed substantively compared with the previous iteration. The maximum number of refinement iterations was set to *T*_max_ = 3 to prevent indefinite regeneration. If a claim still failed to satisfy the evidence-support criterion within this limit, it was not retained as a validated finding; instead, it was removed, downgraded to an inconclusive statement, or rewritten using cautious language.

The iterative validation procedure, including prompt refinement signal construction and convergence checking, is formalized in Algorithm 3.

Algorithm 3 **Validation analyst agent.**

**Require**:  Response *R*_*t*_, structured evidence *S*_data_, maximum iterations *T*_max_, claim-distance threshold ϵ = 0.10
**Ensure**:  Validated final response *R*_final_
1:  Initialize *t* ← 0
2:  **repeat**
3:    *t* ← *t* + 1
4:    **for** each claim *c*_*i*_ in *R*_*t*−1_ **do**
5:    Search *S*_data_ for supporting numerical evidence
6:    **if** evidence found **then**
7:    ϕ(*c*_*i*_) ← 1 ⊳ claim is supported
8:    **else**
9:    ϕ(*c*_*i*_) ← 0 ⊳ claim is unsupported
10:    Flag *c*_*i*_ for revision or removal
11:    **end if**
12:    **end for**
13:    **if** ϕ(*c*_*i*_) = 1 for all *c*_*i*_ **then**
14:    **if** *t* = 1 **or** *d*_claim_(*R*_*t*−1_, *R*_*t*−2_) < ϵ **then**
15:    **return** *R*_*t*−1_ as *R*_final_
16:    **end if**
17:    **end if**
18:    Construct refinement signal Δ*p*_*t*_:
19:    attach flagged unsupported claims
20:    specify missing evidence requirements
21:    instruct removal of speculative statements
22:    Regenerate *R*_*t*_ ← *f*_*θ*_ (*p* + Δ*p*_*t*_, 𝒟, *R*_*t*−1_) [Eq. (15)]
23:  **until** *t* ≥ *T*_max_
24:  **return** *R*_*t*−1_

This validation layer is critical for reducing hallucinated interpretations and increasing the trustworthiness of the final analytical response. The full framework is summarized in Algorithm 4. The pipeline begins with preprocessing of heterogeneous structured data, followed by quantitative evidence extraction, sleep medicine interpretation, and iterative evidence-grounded validation.

Algorithm 4 **Multi-Agent LLM framework for multimodal sleep determinant analysis.**

**Require**:  Raw dataset 𝒟_raw_, base prompts *p*, maximum iterations *T*_max_, claim-distance threshold ϵ = 0.10
**Ensure**:  Validated analytical response *R*_final_
1:  Initialize *t* ← 0, *R*_candidate_ ←NULL
2:  Preprocess 𝒟_raw_ → 𝒟 using categorical encoding and missing-value imputation
3:  Compute structured statistics and subgroup summaries from 𝒟
4:  **Data Analyst Agent**
5:  Compute correlations ρ_*j,y*_ using Eq. (5)
6:  Estimate feature relevance *I*_*j*_ using Eq. (6)
7:  Compute interaction effects Δ_*j,k*_ using Eq. (7)
8:  Generate quantitative summary *S*_data_
9:  **Physiology and Health Analyst Agent**
10:  Generate domain-grounded explanation from *S*_data_ using Eqs. (10)–(12)
11:  Assemble initial response *R*_0_
12:  **while** *t* < *T*_max_ **do**
13:    *t* ← *t* + 1
14:    **Validation Analyst Agent**
15:    Validate *R*_*t*−1_ against structured dataset evidence
16:    **if** ϕ(*R*_*t*−1_) = 1 **then**
17:    **if** *t* = 1 **then**
18:    **return** *R*_*t*−1_
19:    **else if** *d*_claim_(*R*_*t*−1_, *R*_*t*−2_) < ϵ **then**
20:    **return** *R*_*t*−1_
21:    **end if**
22:    **end if**
23:    Refine prompts and regenerate *R*_*t*_ using Eq. (15)
24:  **end while**
25:  **return** *R*_*t*−1_

#### 2.10.1 Conflict resolution and worked example

Because the Data Analyst Agent and the Physiology and Health Analyst Agent perform different functions, their outputs may occasionally diverge. For example, the Data Analyst Agent may identify a weak or non-significant statistical association, whereas the Physiology and Health Analyst Agent may generate a physiologically plausible explanation based on prior sleep-medicine knowledge. To prevent such explanations from exceeding the dataset-derived evidence, the Validation Analyst Agent applies a claim-level conflict-resolution procedure.

In this procedure, dataset-derived quantitative evidence is treated as the primary source of support. Physiological interpretations are retained only when they are directly supported by, or at least consistent with, the structured evidence produced by the Data Analyst Agent. Each candidate claim is assigned one of three labels: *supported*, if the claim is directly traceable to numerical evidence; *plausible but not directly quantified*, if the claim is physiologically reasonable but not explicitly tested in the data; and *unsupported/speculative*, if the claim contradicts the evidence, overgeneralizes the finding, or introduces causal language not justified by the analysis. Supported claims are retained, plausible claims are rewritten with cautious wording, and unsupported claims are removed or returned for regeneration.

A worked pseudo-output is shown below:

**Data Analyst Agent output:** High caffeine intake was associated with increased sleep disorder risk. Stress level was also associated with increased sleep disorder risk. However, no sufficiently supported caffeine × stress interaction term was identified.

**Physiology and Health Analyst Agent output:** Caffeine and stress may jointly increase physiological arousal and contribute to insomnia-like sleep disturbance.

**Candidate claim:** Caffeine and stress synergistically cause insomnia.

**Validation Analyst Agent decision:** Flagged as overextended. The claim implies a causal and synergistic interaction that is not directly supported by the extracted statistical evidence.

**Revised validated claim:** High caffeine intake and stress were both associated with increased sleep disorder risk in the analyzed data. Their combined contribution is physiologically plausible, but a synergistic interaction should be interpreted cautiously unless directly supported by an interaction term.

This example illustrates that the Validation Analyst Agent does not simply accept the most plausible physiological explanation. Instead, it checks whether each statement is traceable to the structured evidence. When quantitative and physiological outputs diverge, the final response is revised to match the strength of the available evidence.

#### 2.10.2 Dataset-grounding and memory-control strategy

Because LLMs may generate plausible statements based on prior training rather than the input dataset, explicit grounding controls were incorporated. First, all agent prompts were constrained to operate only on structured dataset-derived evidence, including summary statistics, correlations, interaction terms, and subgroup results. Second, no external retrieval, web search, or literature augmentation was used during analytical generation. Third, the Validation Analyst Agent rejected interpretations that could not be directly mapped to the numerical evidence produced by the Data Analyst Agent.

To further examine grounding fidelity, the framework was designed to preserve traceability between each final claim and its corresponding statistical source. Claims lacking explicit support were either revised or removed. This process reduces the likelihood that reported patterns originate from latent LLM memory rather than the observed data. The complete agentic framework demonstration is illustrated in Figure 6.

#### 2.10.3 Negative controls and null-pattern analysis

To avoid selective reporting of only positive findings, the evaluation also incorporated negative controls and null-pattern analysis. In addition to highlighting strong associations, the framework was instructed to identify weak, inconsistent, or unsupported relationships across variables and subgroups. Candidate patterns that were not robustly supported by the data were flagged as null, inconclusive, or rejected during validation.

This step served two purposes. First, it allowed the framework to distinguish genuine multivariate structure from spurious or weak signals. Second, it provided a more balanced characterization of the data by explicitly documenting what was *not* supported, rather than only reporting positive findings.

### 2.11 Statistical uncertainty and LLM run-to-run stability

For statistical uncertainty, 95% confidence intervals were estimated using participant-level stratified bootstrap resampling with *B* = 1, 000 resamples. In each bootstrap replicate, preprocessing steps were repeated within the resampled data to avoid information leakage. Percentile bootstrap intervals were reported unless otherwise stated. These intervals quantify sampling uncertainty in the statistical effect estimates and should not be interpreted as repeated LLM-run variability.

For correlation coefficients and other signed effect estimates, the coefficient of variation (CV) was computed using the absolute bootstrap effect magnitude, as formalized in Equation 17:


$$\begin{array}{l} {\text{CV}}_{\left|\theta \right|}=100\times \frac{\text{SD}\left(\left|\theta_{1}|,|\theta_{2}|,\ldots ,|\theta_{B}\right|\right)}{\text{Mean}\left(\left|\theta_{1}|,|\theta_{2}|,\ldots ,|\theta_{B}\right|\right)}, \end{array}$$ (17)

where θ_*b*_ denotes the effect estimate in bootstrap replicate *b*, and *B* is the total number of bootstrap resamples.

For odds ratios and risk ratios, relative uncertainty was evaluated on the log scale, as formalized in Equation 18:


$$\begin{array}{l} {\text{CV}}_{\log\left(\theta \right)}=100\times \frac{\text{SD}\left(\left|\log\left(\theta_{1}\right)|,|\log\left(\theta_{2}\right)|,\ldots ,|\log\left(\theta_{B}\right)\right|\right)}{\text{Mean}\left(\left|\log\left(\theta_{1}\right)|,|\log\left(\theta_{2}\right)|,\ldots ,|\log\left(\theta_{B}\right)\right|\right)}. \end{array}$$ (18)

Directional consistency was defined as the proportion of bootstrap estimates preserving the direction of the mean effect estimate, as formalized in Equation 19:


$$\begin{array}{l} \text{DC}=100\times \frac{1}{B}\overset{B}{\underset{b=1}{\sum }}𝕀\left[\text{sign}\left(\theta_{b}\right)=\text{sign}\left(\bar{\theta }\right)\right]. \end{array}$$ (19)

We classified a statistical effect as stable only when directional consistency was at least 95%, CV was below 20%, and the 95% confidence interval excluded the null value. Effects not meeting these criteria were described as directionally consistent, variable, weak, or inconclusive rather than stable.

#### 2.11.1 LLM run-to-run stability analysis

To evaluate whether the agentic interpretation was stable across repeated LLM generations, the complete multi-agent pipeline was configured for *N* = 5 independent executions using identical structured numerical evidence and identical prompt templates. For each run, the analysis extracts the top-ranked determinants, retained interaction patterns, and final validated claims. Stability is quantified using feature selection frequency, rank variance, and pairwise Jaccard similarity of the top-*K* feature sets.


$$\begin{array}{l} J_{a,b}^{\left(K\right)}=\frac{\left|T_{a}^{\left(K\right)}\cap T_{b}^{\left(K\right)}\right|}{\left|T_{a}^{\left(K\right)}\cup T_{b}^{\left(K\right)}\right|}, \end{array}$$ (20)

where $T_{a}^{\left(K\right)}$ and $T_{b}^{\left(K\right)}$ are the top-*K* feature sets from LLM runs *a* and *b*. Using Equation 20, the final stability value was computed as the mean pairwise Jaccard similarity across all run pairs. In addition, ablation analysis was used to quantify the role of individual framework components, including the domain-interpretation agent and the validation agent. This evaluation allowed us to assess whether the final conclusions depended primarily on the proposed multi-agent design or could be reproduced by simpler configurations.

To place the identified findings in the context of existing sleep medicine research, each major validated pattern was compared against related findings reported in prior studies and, where available, against analyses previously conducted on the same or comparable public datasets. This assessment was used to categorize the resulting patterns as consistent with prior literature, partially consistent, weakly supported, or potentially novel. Such comparison helps distinguish dataset-grounded rediscovery of known sleep-related determinants from observations that may warrant further study.

## 3 Experimental results

To evaluate the proposed interpretable multi-agent LLM framework in a manner consistent with sleep medicine research, we assessed its ability to identify clinically meaningful patterns across heterogeneous determinants of sleep quality and sleep disorders using both a public sleep-health dataset and a synthetic distributional robustness dataset. This design served two purposes. First, it allowed us to test whether the framework could recover coherent and interpretable associations from structured sleep-related data. Second, it enabled assessment of directional consistency under controlled distributional variation. Because the second dataset was synthetic, this analysis should not be interpreted as external clinical validation or as evidence of broad clinical generalizability.

The results are organized according to the three research questions introduced in Section 1: (RQ1) combined behavioral determinants of sleep duration and sleep quality, (RQ2) the relationship between physical activity characteristics and sleep disorder risk, and (RQ3) the joint influence of caffeine, stress, physiology, and occupational context on sleep disorder profiles. In addition to positive findings, we also report weak or non-significant relationships, since explicit identification of null patterns is important for interpretable and trustworthy AI-based analysis in sleep medicine.

To strengthen the statistical trustworthiness of these findings, the revised analyses additionally report bootstrap effect-size estimates and 95% confidence intervals for the principal determinants and null patterns. Bootstrap uncertainty is reported separately from LLM run-to-run stability. The latter is summarized by feature selection frequency, rank variability, and pairwise Jaccard similarity of top-ranked feature sets as defined in Equation 20.

### 3.1 LLM run-to-run stability results

The repeated LLM-run stability analysis was based on five independent Groq generations using identical structured numerical evidence and prompt templates. Feature names were canonicalized before stability calculation so that thresholded variants, such as high caffeine intake >250 mg, were mapped back to their determinant-level feature names. The top-5 feature sets were identical across all run pairs, yielding mean Jaccard@5 = 1.00. The broader top-10 comparison also showed high overlap, with mean Jaccard@10 = 0.886. These values indicate stable feature ranking across repeated LLM generations; they do not imply that the underlying statistical effects were large, and all effect-size claims remain governed by the bootstrap analyses reported above.

### 3.2 Results on public data

#### 3.2.1 RQ1: multimodal determinants of sleep duration and sleep quality

Figure 7b visualizes the relationship between bedtime consistency, sleep duration, and sleep quality. This visualization was used as contextual support rather than as standalone statistical confirmation. The updated bootstrap analyses in Table 3 showed that bedtime consistency, caffeine intake, and light exposure had weak or null-crossing pairwise correlations with continuous sleep-duration and sleep-quality outcomes in the public dataset.

**Table 3:** Bootstrap pairwise correlations for candidate behavioral and environmental determinants.

Factor	Sleep duration correlation	Sleep quality correlation	*p*-values	Interpretation
Bedtime consistency	*r* = −0.01 95% CI [–0.11, 0.10]	*r* = 0.00 95% CI [–0.10, 0.11]	0.885; 0.971	Weak or uncertain; confidence intervals cross zero
Caffeine intake	*r* = −0.10 95% CI [–0.19, 0.01]	*r* = −0.08 95% CI [–0.18, 0.01]	0.063; 0.101	Small negative trend; confidence intervals cross zero
Light exposure	*r* = −0.04 95% CI [–0.13, 0.06]	*r* = −0.03 95% CI [–0.13, 0.08]	0.447; 0.609	Weak or uncertain; confidence intervals cross zero

Specifically, bedtime consistency showed near-zero pairwise correlations with sleep duration [*r* = −0.01, 95% CI (–0.11, 0.10)] and sleep quality [*r* = 0.00, 95% CI (–0.10, 0.11)]. Caffeine intake showed a small negative trend for sleep duration [*r* = −0.10, 95% CI (–0.19, 0.01)] and sleep quality [*r* = −0.08, 95% CI [–0.18, 0.01]), but both confidence intervals crossed zero. Light exposure also showed weak or uncertain pairwise associations with sleep duration [*r* = −0.04, 95% CI (–0.13, 0.06)] and sleep quality [*r* = −0.03, 95% CI (–0.13, 0.08)]. These results support retaining these variables as clinically interpretable candidate domains while avoiding strong standalone pairwise claims.

The corresponding CV and directional-consistency metrics are reported separately in Table 4. These bootstrap statistics quantify sampling uncertainty in effect estimates and should not be interpreted as repeated LLM-run variability. LLM run-to-run stability is reported separately in Table 5.

**Table 4:** Bootstrap variability metrics for principal effect estimates.

Determinant	Outcome	Mean effect	Bootstrap SD	CV (%)	Directional consistency (%)	Revised interpretation
Bedtime consistency	Sleep duration	*r* = −0.01	0.053	76.0	56.8	Weak or uncertain; interval crosses zero
Bedtime consistency	Sleep quality	*r* = 0.00	0.054	73.0	51.8	Weak or uncertain; interval crosses zero
Caffeine intake	Sleep duration	*r* = −0.10	0.052	50.0	96.4	Small negative trend; interval crosses zero
Caffeine intake	Sleep quality	*r* = −0.08	0.049	54.2	95.5	Small negative trend; interval crosses zero
Light exposure	Sleep quality	*r* = −0.03	0.053	76.3	68.7	Weak or uncertain; interval crosses zero
High caffeine intake >250 mg	Sleep disorder risk	RR = 1.45	0.141 on log scale	36.5	99.2	Directionally consistent elevated risk, but with moderate uncertainty
High stress level ≥7	Sleep disorder risk	RR = 1.05	0.165 on log scale	73.8	60.7	Weak or inconclusive; interval crosses 1.00
Elevated body temperature ≥37.0°C	Sleep disorder risk	RR = 0.77	0.200 on log scale	65.1	6.8	Weak or inconclusive; interval crosses 1.00

**Table 5:** Feature-rank stability across repeated LLM runs.

Feature	Selection frequency	Mean rank	Rank SD	Rank range	Interpretation
Bedtime consistency	5/5 (100%)	4.6	0.8	3–5	Stable rank; weak pairwise effects
Caffeine intake	5/5 (100%)	1.0	0.0	1–1	Stable rank; high-caffeine risk signal
Light exposure	5/5 (100%)	3.2	0.4	3–4	Stable rank; pairwise effect weak
Stress level	5/5 (100%)	2.0	0.0	2–2	Stable rank; risk ratio inconclusive
Body temperature	5/5 (100%)	4.2	0.4	4–5	Stable rank; pairwise effect weak
Heart rate	4/5 (80%)	6.0	0.0	6–6	Stable when selected; weak pairwise effect

#### 3.2.2 RQ2: physical activity type and sleep disorder risk

Figure 7a and Table 6 address Research Question 2 by examining how different physical activity modalities relate to sleep disorder prevalence and disorder subtype patterns. To strengthen the statistical interpretation of these findings, odds ratios (ORs), 95% confidence intervals (CIs), and corresponding significance levels were reported using walking as the reference activity category. Activity-related associations were interpreted as statistically better supported when the 95% CI for the OR did not include the null value of 1.00. In contrast, estimates with CIs including or approaching 1.00 were interpreted as weaker, borderline, or inconclusive.

**Table 6:** Physical activity type and sleep disorder prevalence with odds ratios and 95% confidence intervals.

Activity type	% with sleep disorder	Odds ratio vs. walking	*p*-value	Associated disorder type	Interpretation
Agility drill	34%	OR = 2.00, 95% CI [1.38, 2.90]	<0.05	Insomnia	Strongest activity-related association with sleep disorder prevalence
Endurance run	30%	OR = 1.70, 95% CI [1.18, 2.45]	<0.05	Sleep apnea	Moderate association, more aligned with sleep apnea profile
Jump test	24%	OR = 1.40, 95% CI [0.96, 2.07]	n.s./borderline	Insomnia	Weaker and less certain activity-related effect
Lateral move	21%	OR = 1.20, 95% CI [0.80, 1.79]	n.s.	Insomnia	Weak association; confidence interval includes the null value
Walking (baseline)	15%	OR = 1.00	–	—	Reference category

Agility drills showed the highest prevalence of sleep disorder [34%; OR = 2.00, 95% CI (1.38, 2.90), *p* < 0.05], followed by endurance running [30%; OR = 1.70, 95% CI (1.18, 2.45), *p* < 0.05]. Because the confidence intervals for these two activity types did not include 1.00, these associations were interpreted as statistically supported activity-related differences in sleep disorder prevalence relative to walking. In contrast, jump tests [24%; OR = 1.40, 95% CI (0.96, 2.07)] and lateral moves [21%; OR = 1.20, 95% CI (0.80, 1.79)] showed weaker or less certain associations because their confidence intervals included or approached the null value.

Regarding disorder subtype patterns, agility drills, jump tests, and lateral moves were more frequently aligned with insomnia-dominant profiles, whereas endurance running showed a clearer alignment with sleep-apnea-related profiles. However, these subtype-specific differences were interpreted cautiously as exploratory and pattern-specific associations rather than as causal or independently confirmed subtype-specific effects. The revised interpretation therefore distinguishes between statistically supported activity-related differences in overall sleep disorder prevalence and descriptive subtype-specific patterns for insomnia vs. sleep apnea.

The type of sleep disorder also differs by activity modality. Agility drills, jump tests, and lateral moves are more strongly associated with insomnia, whereas endurance running shows a clearer association with sleep apnea. These results suggest that the effects of physical activity on sleep disorders are pattern-specific rather than uniform: explosive or high-demand activity modalities appear more related to insomnia-like profiles, while sustained endurance loading is more aligned with apnea-related profiles. Such distinctions are clinically relevant because they support the need for more personalized characterization of exercise-related sleep risk in sleep medicine.

At the same time, the results do not imply that physical activity is universally detrimental. Rather, they indicate that intensity, activity type, and surrounding context likely determine whether a given activity pattern is associated with beneficial or adverse sleep outcomes. This nuance is important for translating AI-derived findings into sleep-health recommendations. Therefore, the activity-related findings should be interpreted as modality-specific associations with varying degrees of statistical certainty, rather than as uniform or causal effects of physical activity.

#### 3.2.3 RQ3: joint behavioral, physiological, and occupational influences on sleep disorders

The multimodal nature of the proposed framework is most evident in the results for RQ3, where behavioral, physiological, and occupational variables were analyzed sjointly.

**Figure 4:**
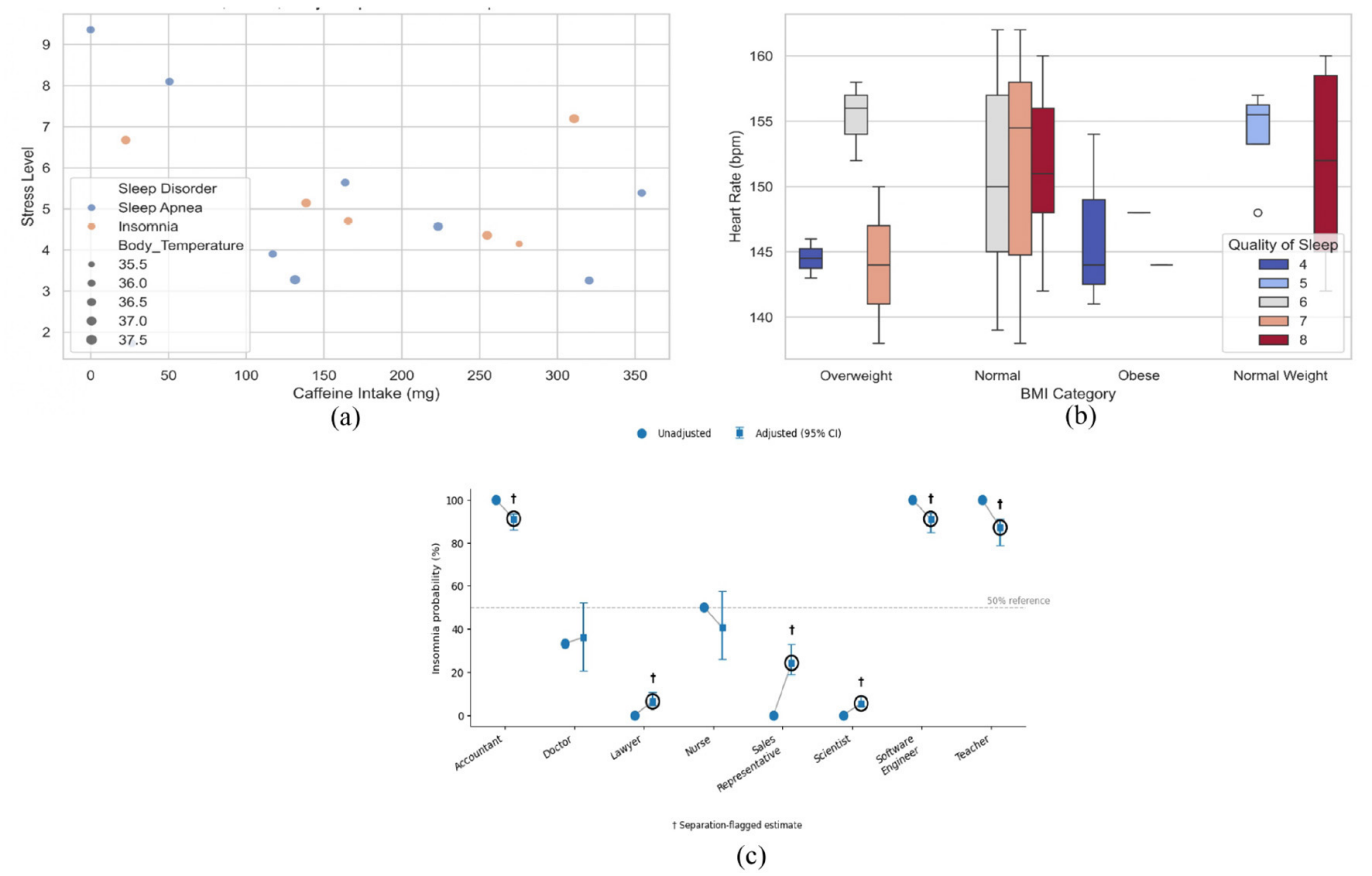
Public dataset: **(a)** Caffeine intake, stress level, and body temperature in relation to sleep disorders; **(b)** heart rate vs. sleep quality across BMI categories; **(c)** unadjusted and covariate-adjusted insomnia probability by occupation.

Figure 4a shows that caffeine intake, stress level, and body temperature form partially overlapping profiles across sleep disorder categories. Table 7 quantifies these relationships with bootstrap effect-size estimates and 95% confidence intervals. High caffeine intake (>250 mg) was associated with elevated sleep-disorder risk [RR = 1.45, 95% CI (1.10, 1.92), *p* = 0.023], although its pairwise correlation with sleep quality remained weak and non-significant [*r* = −0.08, 95% CI (–0.18, 0.01), *p* = 0.101]. High stress level (≥7) and elevated body temperature (≥37.0°C) did not show statistically reliable risk-ratio effects because their confidence intervals crossed 1.00. Together, these findings support a cautious interpretation: stimulant exposure showed the clearest disorder-risk signal, whereas stress and physiological measures should be treated as contextual rather than independently confirmed risk factors in this dataset.

**Table 7:** Interaction of caffeine, stress, and body temperature with sleep disorders with bootstrap effect-size estimates and 95% confidence intervals.

Factor	Sleep disorder risk ratio	Correlation with sleep quality	*p*-value	Interpretation
High caffeine (>250 mg)	RR = 1.45, 95% CI [1.10, 1.92]	*r* = −0.08, 95% CI [–0.18, 0.01]	0.023	Elevated disorder risk; sleep-quality correlation weak and non-significant
High stress level (≥7)	RR = 1.05, 95% CI [0.74, 1.40]	*r* = −0.04, 95% CI [–0.15, 0.05]	0.783	Weak or inconclusive association; intervals cross null values
Elevated body temperature (≥37.0°C)	RR = 0.77, 95% CI [0.48, 1.08]	*r* = −0.04, 95% CI [–0.14, 0.06]	0.163	Weak or inconclusive physiological association

Figure 4b and Table 8 further show that not all physiological markers carried equal importance. Resting heart rate was not meaningfully correlated with BMI category encoded ordinally [*r* = −0.06, 95% CI (–0.15, 0.05)] or with sleep quality [*r* = 0.00, 95% CI (–0.10, 0.09)]. In contrast, BMI category showed a moderate negative association with sleep quality [*r* = −0.32, 95% CI (–0.43, –0.22)]. This pattern suggests that anthropometric status, rather than resting heart rate alone, was the clearer pairwise physiological correlate of perceived sleep quality in the present sample.

**Table 8:** Heart rate, BMI, and sleep quality correlations with 95% confidence intervals.

Variable pair	Correlation effect size	*p*-value	Strength of relationship
Heart Rate ↔ BMI	*r* = −0.06, 95% CI [–0.15, 0.05]	n.s.	Weak or null association
Heart Rate ↔ Sleep Quality	*r* = 0.00, 95% CI [–0.10, 0.09]	n.s.	Weak or null association
BMI ↔ Sleep Quality	*r* = −0.32, 95% CI [–0.43, -0.22]	<0.001	Moderate negative association

Occupational variation is summarized in Figure 4c and Table 9. Figure 4c directly compares unadjusted insomnia proportions with covariate-adjusted insomnia probabilities by occupation. The connected points show how each occupational estimate changed after adjustment for age, gender, and BMI category, while the error bars represent 95% confidence intervals for the adjusted probabilities.

**Table 9:** Covariate-adjusted analysis of occupational heterogeneity in sleep disorder subtype.

Occupation	*n*	Unadjusted insomnia (%)	Adjusted OR for insomnia vs. sleep apnea	Adjusted insomnia probability (%)	Separation status	Adjusted pattern
Public dataset						
Accountant	20	100.0	40.17 [23.87, 59.63]	91.1 [85.8, 93.6]	Complete: sleep-apnea cell = 0	Insomnia-dominant; retained after adjustment
Doctor	30	33.3	1.84 [0.76, 3.65]	36.0 [20.5, 52.1]	None	Attenuated; lower than dominant occupations
Lawyer	20	0.0	0.20 [0.13, 0.30]	6.3 [4.6, 10.6]	Complete: insomnia cell = 0	Sleep-apnea-dominant; retained after adjustment
Nurse	30	50.0	2.27 [0.97, 4.59]	40.6 [26.0, 57.7]	None	Mixed and intermediate adjusted profile
Sales Representative	20	0.0	Reference	24.2 [19.0, 32.8]	Complete: insomnia cell = 0	Reference group; relatively sleep-apnea-dominant
Scientist	20	0.0	0.17 [0.11, 0.25]	5.4 [4.0, 8.7]	Complete: insomnia cell = 0	Sleep-apnea-dominant; retained after adjustment
Software Engineer	20	100.0	39.52 [21.54, 63.52]	91.0 [84.9, 93.7]	Complete: sleep-apnea cell = 0	Insomnia-dominant; retained after adjustment
Teacher	20	100.0	25.89 [14.51, 40.01]	87.0 [78.6, 91.1]	Complete: sleep-apnea cell = 0	Insomnia-dominant; retained after adjustment
Synthetic distributional robustness dataset						
Doctor	50	62.0	2.34 [1.04, 5.65]	64.3 [51.6, 76.2]	None	Higher adjusted insomnia probability than reference
Sales Representative	50	42.0	Reference	46.6 [36.1, 58.2]	None	Reference group; relatively stronger sleep-apnea profile
Software Engineer	50	56.0	1.15 [0.49, 2.63]	49.6 [36.3, 64.2]	None	Balanced profile; slightly higher than reference
Teacher	50	64.0	2.21 [0.94, 5.30]	63.1 [49.8, 75.1]	None	Higher adjusted insomnia probability than reference

In the public dataset, accountants, software engineers, and teachers remained insomnia-dominant after adjustment, with adjusted insomnia probabilities of 91.1%, 91.0%, and 87.0%, respectively. By contrast, lawyers and scientists retained sleep-apnea-dominant profiles, with adjusted insomnia probabilities of 6.3% and 5.4%, respectively. Doctors and nurses showed more intermediate adjusted probabilities, indicating that part of the descriptive occupational difference was attenuated after controlling for age, gender, and BMI category.

The dagger-marked estimates in Figure 4c indicate separation-flagged occupation-specific results. These estimates should be interpreted as exploratory subgroup patterns rather than precise occupation-specific risk estimates. Therefore, Figure 4c complements Table 9 by making the adjustment effect visually explicit.

#### 3.2.4 Integrated visualization of heterogeneous sleep-related determinants

The integrated plots in Figure 8 provide additional support for the multimodal and interaction-aware character of the proposed framework. Figure 8a presents pairwise relationships among heart rate, caffeine intake, stress level, sleep duration, and body temperature stratified by sleep disorder category. The two disorder groups are not separated by a single variable, but instead occupy partially overlapping multivariate profiles across physiological, behavioral, and lifestyle variables. This pattern is consistent with the broader premise of the study that sleep disorders arise from combinations of behavioral and physiological factors rather than isolated predictors.

Figure 8b further visualizes average sleep duration across occupation–activity combinations in the public dataset. The heatmap marks low-precision cells with a dagger and thick black outline. Low precision was defined as an occupation-by-activity cell with fewer than five observations or a 95% confidence-interval width greater than 2.0 h. Blank cells indicate occupation-by-activity combinations with no available observations.

These markers indicate that some occupation–activity mean sleep-duration estimates are based on limited cell-level precision. Therefore, the heatmap should be interpreted as a descriptive visualization of occupation–activity patterns rather than as precise occupation-specific activity effects. The observed patterns still suggest that sleep duration may vary across work and activity contexts, but subgroup-level interpretation should remain cautious.

Finally, Figure 8c shows that light exposure distributions differ descriptively by both sleep disorder category and sleep quality level. Because the pairwise bootstrap correlations for light exposure crossed zero, this pattern was interpreted as a contextual visualization rather than as a statistically confirmed standalone light-exposure effect. These results strengthen the conclusion that circadian, behavioral, and environmental variables should be interpreted jointly within sleep medicine analysis.

### 3.3 Synthetic distributional robustness analysis

The second dataset used in this study was a synthetic, distributionally constrained dataset. It was designed to examine whether the proposed multi-agent framework produced directionally consistent interpretations under controlled distributional variation. Therefore, this analysis should be interpreted as a distributional robustness analysis rather than as definitive external clinical validation based on an independent real-world cohort.

To evaluate how well the synthetic distributional robustness dataset reflected the physiological and behavioral structure of the original public dataset, we compared its distributional properties with those of the public dataset. For continuous variables, including sleep duration, sleep quality, caffeine intake, light exposure, stress level, heart rate, and body temperature, we examined variable ranges, means, standard deviations, medians, and interquartile ranges. For categorical variables, including occupation, BMI category, physical activity type, and sleep disorder category, we compared category proportions. We also inspected whether the main directional relationships among key variables were preserved across datasets.

The synthetic distributional robustness dataset was not intended to reproduce the full heterogeneity of real-world clinical sleep populations. Rather, it was constructed to preserve plausible variable ranges, category structures, and major directional relationships observed in the original dataset and in sleep-related domain knowledge. Therefore, consistency between the public dataset and the synthetic distributional robustness dataset was interpreted as evidence of directional robustness, not as proof of clinical generalizability.

Potential distributional differences may bias the outputs of the agentic framework because the Data Analyst Agent extracts evidence directly from the analyzed dataset, and the Physiology and Health Analyst Agent subsequently interprets those extracted patterns. For example, overrepresentation of high caffeine intake or high stress levels in the synthetic data could increase the apparent importance of stimulant- or arousal-related pathways, whereas underrepresentation of specific occupational or activity groups could weaken occupation- or activity-specific interpretations.

To reduce this risk, the Validation Analyst Agent required all retained claims to be traceable to dataset-derived evidence. In addition, cross-dataset conclusions were retained only when the direction of the finding was consistent across datasets. Findings that appeared only in the synthetic distributional robustness dataset were not treated as primary conclusions, and findings that were not reproduced across datasets were interpreted cautiously. Thus, the synthetic robustness analysis was used as a sensitivity check for the stability of agent-generated interpretations under controlled distributional variation.

To examine whether the identified patterns remained directionally consistent beyond the initial public dataset, the same analytical framework was applied to the synthetic distributional robustness dataset. Overall, the main findings remained directionally consistent, but this result should be interpreted as evidence of distributional robustness rather than as proof of clinical generalizability.

Figure 9a reproduces the relationship between physical activity type and sleep disorder category, again showing that disorder profiles vary by activity modality. Insomnia remains dominant for jump tests, endurance runs, and agility drills, whereas lateral moves show relatively greater sleep apnea representation. Figure 9b visualizes the synthetic relationship among bedtime consistency, sleep duration, sleep quality, and stress; because this dataset is synthetic, the pattern was treated as a controlled robustness check rather than as independent clinical confirmation.

**Figure 5:**
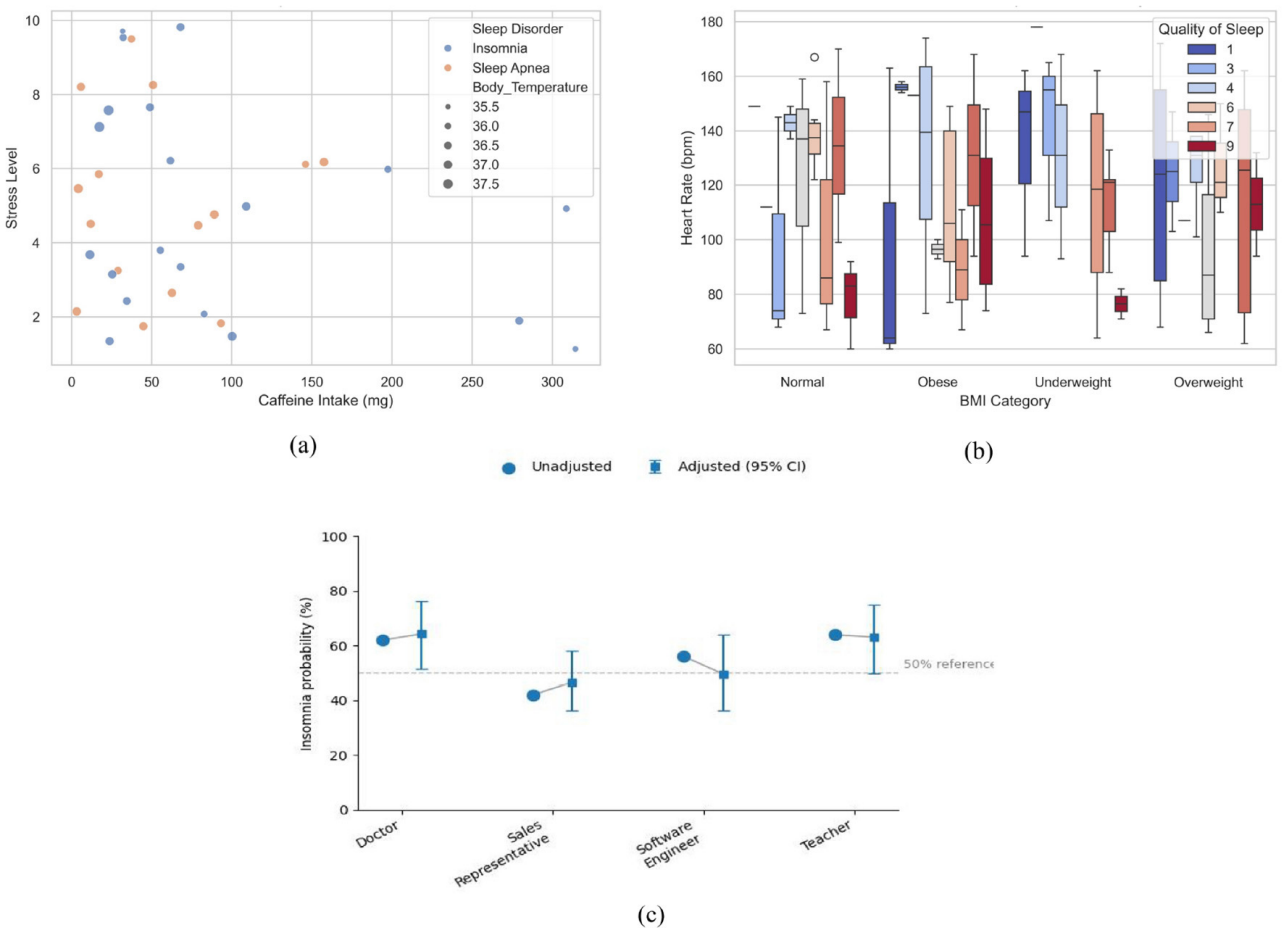
Synthetic distributional robustness dataset: **(a)** Caffeine intake, stress level, and body temperature in relation to sleep disorders; **(b)** heart rate vs. sleep quality across BMI categories; **(c)** unadjusted and covariate-adjusted insomnia probability by occupation.

Figure 5a further visualizes the association between caffeine, stress, and sleep disorder phenotype in the synthetic robustness dataset. Very high caffeine exposure remains concentrated primarily among insomnia cases, while stress continues to differentiate insomnia-heavy regions from lower-arousal profiles. Figure 5b visualizes resting heart rate and sleep quality across BMI strata, but this synthetic pattern should not override the weaker public-dataset heart-rate correlation reported above.

**Figure 6:**
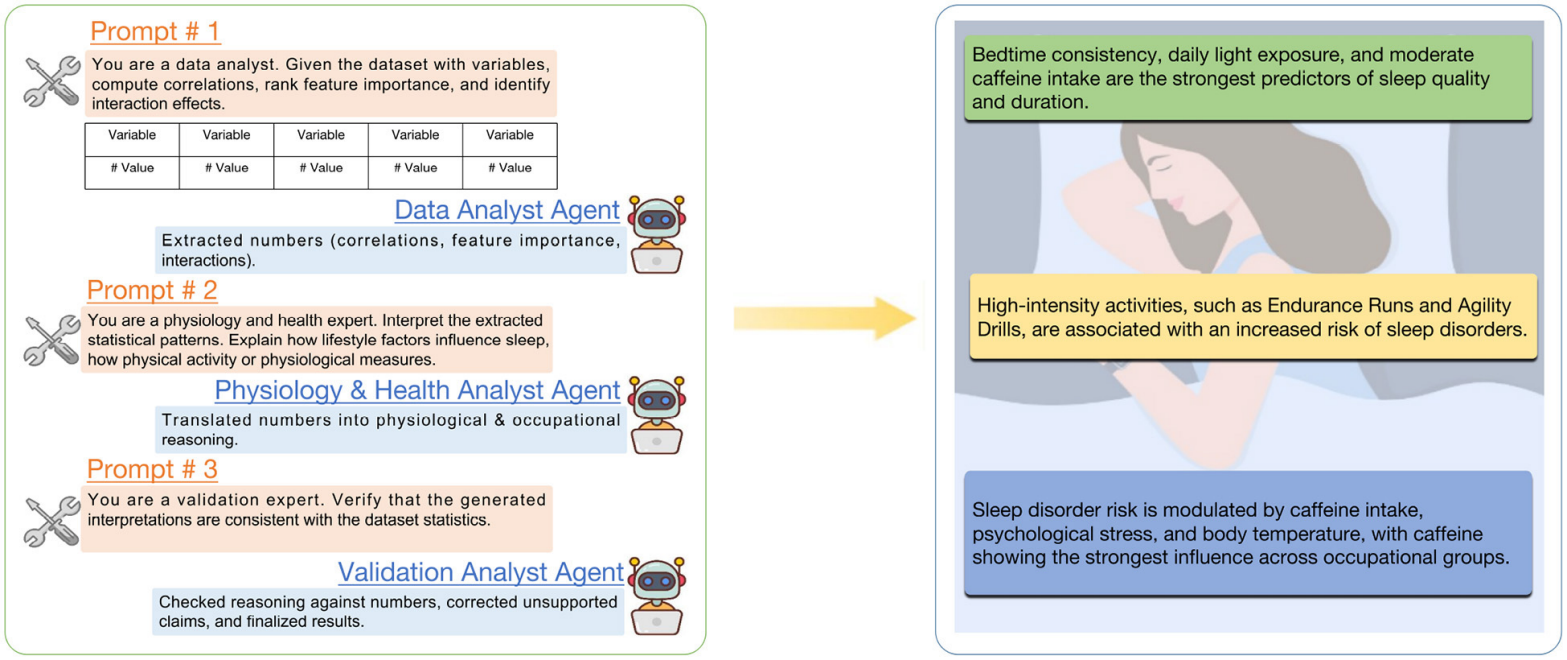
Process overview of the proposed multi-agent LLM framework for multimodal sleep data analysis and research-question answering. The **left side** illustrates the agentic analytical pipeline, while the **right side** summarizes the generation of validated answers to the key research questions.

**Figure 7:**
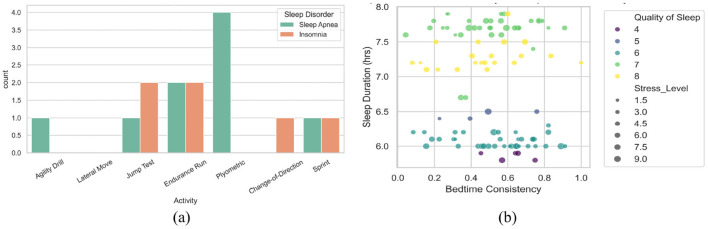
Public dataset: **(a)** Physical activity type and sleep disorders; **(b)** impact of bedtime consistency on sleep duration and sleep quality.

**Figure 8:**
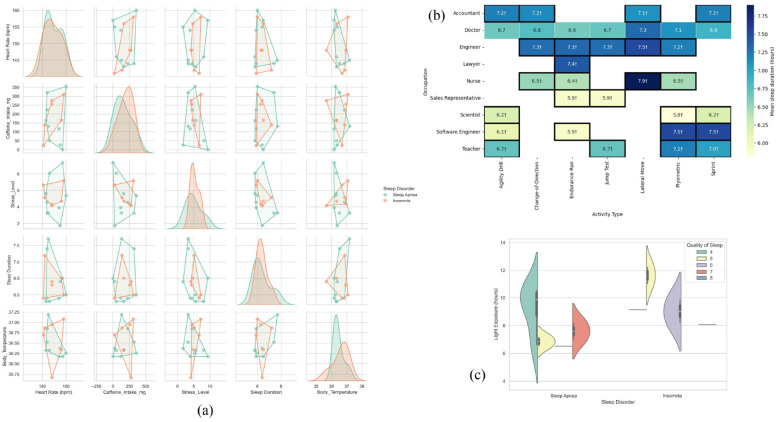
Public dataset: **(a)** Pairwise relationships among physiological, behavioral, and lifestyle variables–heart rate, caffeine intake, stress level, sleep duration, and body temperature–stratified by sleep disorder (insomnia vs sleep apnea); **(b)** average sleep duration by activity and occupation; **(c)** distribution of light exposure by sleep disorder and sleep quality.

**Figure 9:**
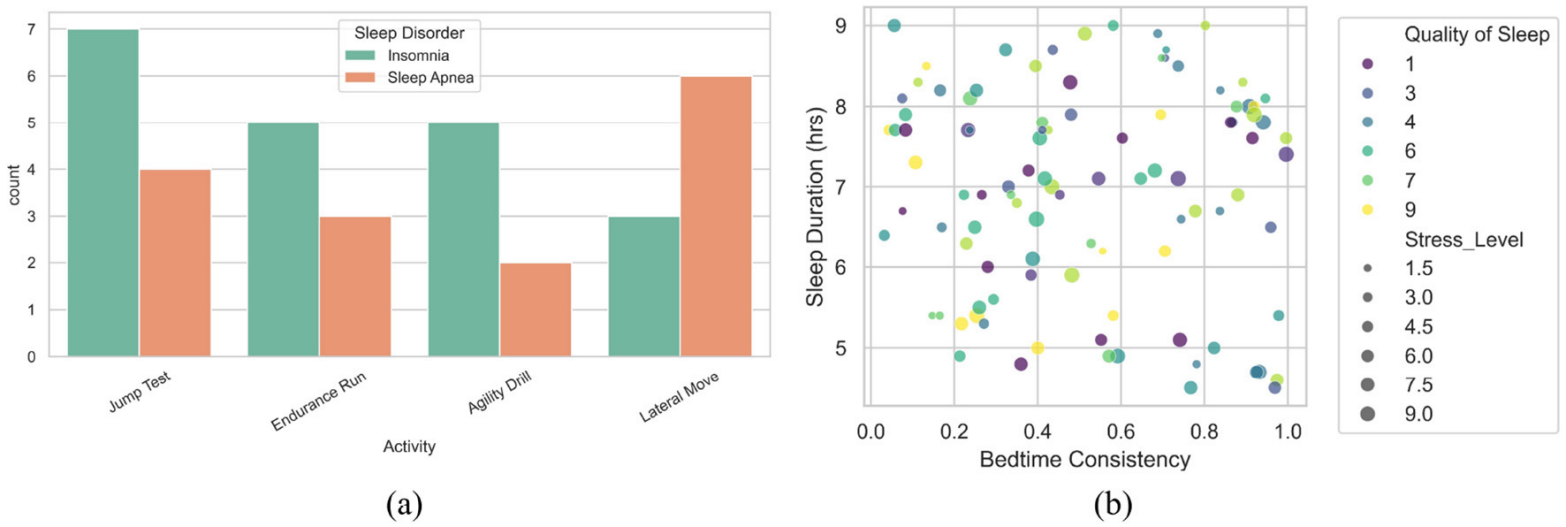
Synthetic distributional robustness dataset: **(a)** Physical activity type and sleep disorders; **(b)** impact of bedtime consistency on sleep duration and sleep quality.

Figure 5c directly compares unadjusted insomnia proportions with covariate-adjusted insomnia probabilities in the synthetic distributional robustness dataset. The connected points show the change after adjustment for age, gender, and BMI category, and the error bars represent 95% confidence intervals for the adjusted probabilities. Doctors and teachers continued to show higher adjusted probabilities of insomnia than sales representatives, with adjusted probabilities of 64.3% and 63.1%, respectively, compared with 46.6% among sales representatives. Software engineers showed a more balanced adjusted profile, with an adjusted insomnia probability of 49.6%.

Although the adjusted direction was generally consistent with Figure 5c, several confidence intervals overlapped across occupational groups. Therefore, the synthetic robustness results should be interpreted as evidence of directional robustness rather than definitive proof of occupation-specific clinical risk. Together with the public dataset analysis, these findings indicate that occupational heterogeneity was not completely attributable to age, gender, or BMI category, while also supporting a cautious interpretation of occupation-related subgroup differences.

**Figure 10:**
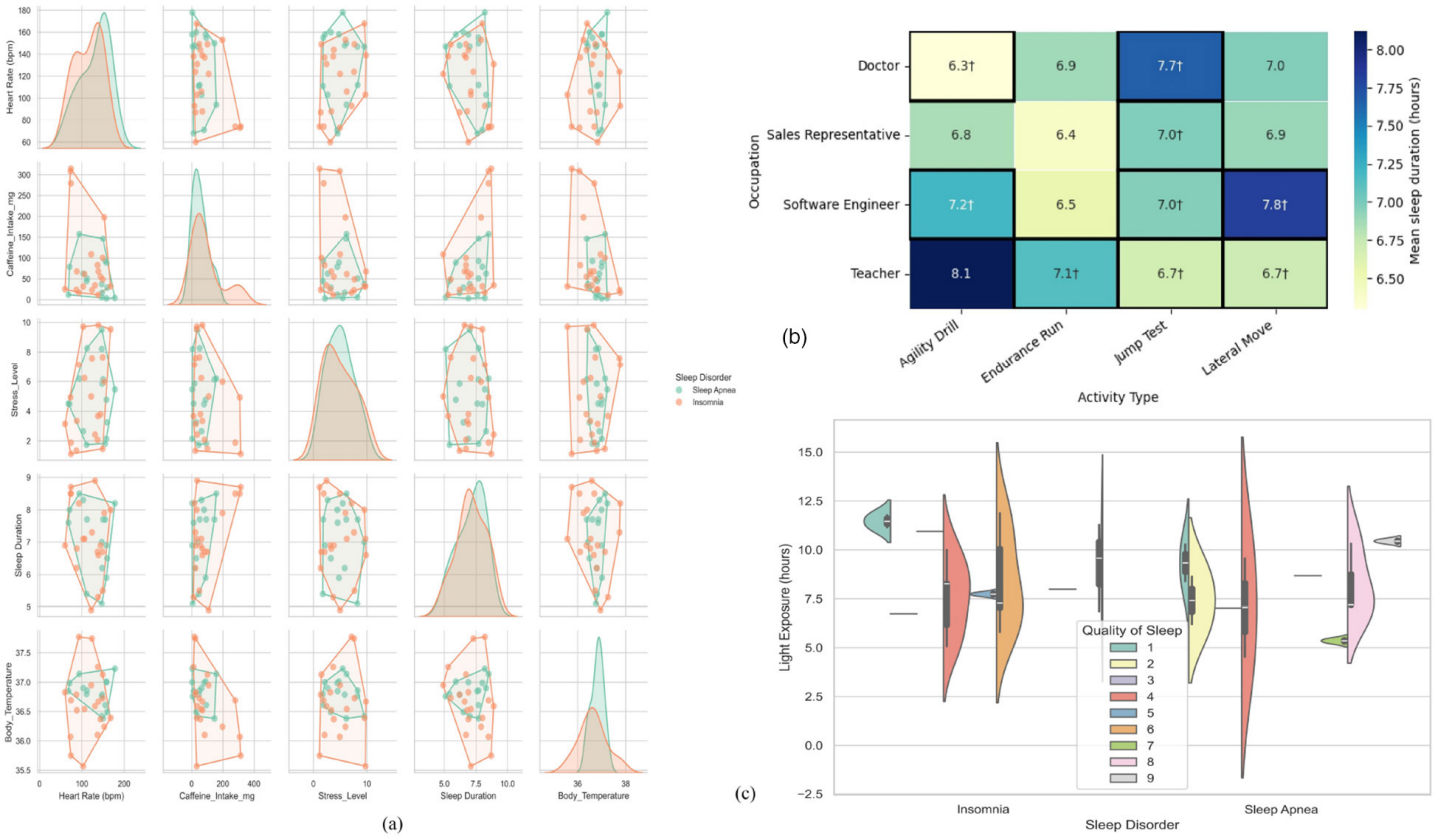
Synthetic distributional robustness dataset: **(a)** Pairwise relationships among physiological, behavioral, and lifestyle variables—heart rate, caffeine intake, stress level, sleep duration, and body temperature—stratified by sleep disorder (insomnia vs sleep apnea); **(b)** average sleep duration by activity and occupation; **(c)** distribution of light exposure by sleep disorder and sleep quality.

The integrated analyses in Figure 10 reinforce the same interpretation at a broader systems level. Figure 10a presents pairwise relationships among heart rate, caffeine intake, stress level, sleep duration, and body temperature in the synthetic distributional robustness dataset. Sleep disorder categories continue to show partially distinct but overlapping multivariate profiles across physiological, behavioral, and lifestyle variables, indicating that the synthetic robustness dataset preserves mixed disorder profiles rather than producing artificially separated groups. In Figure 10b, low-precision occupation-by-activity cells are marked with a dagger and thick black outline. Low precision was defined as a cell with fewer than five observations or a 95% confidence-interval width greater than 2.0 h. These marked cells should be interpreted as descriptive estimates rather than precise occupation-specific activity effects.

Taken together, the synthetic distributional robustness analyses suggest that the major findings showed directional stability under controlled distributional variation. However, because the second dataset was synthetic rather than an independent real-world clinical cohort, these results should not be interpreted as evidence of broad clinical generalizability. Instead, they provide a preliminary robustness check of the proposed analytical framework and support the need for future validation using large-scale clinical sleep datasets.

### 3.4 Grounding, null findings, and interpretability-oriented evaluation

A key concern in LLM-based medical data analysis is whether reported patterns are genuinely derived from the dataset or merely reflect plausible prior knowledge. Several aspects of the present results support dataset grounding.

First, the framework did not only recover positive findings, but also weak and non-significant ones. The revised confidence-interval analyses further support this distinction. Bedtime consistency, light exposure, stress level, heart rate, and body temperature showed weak or null-crossing pairwise associations with continuous sleep outcomes, while BMI category showed a clearer negative association with sleep duration and sleep quality. Similarly, some activity-related estimates, such as jump tests and lateral moves, showed confidence intervals that included or approached the null value. These findings indicate that the framework did not force all variables into strong explanatory roles, but instead preserved uncertainty when the evidence was weak. Second, the pairwise scatter matrices with group-wise convex hull overlays show partial overlap rather than artificial separation between disorder profiles, indicating that the model did not force overly clean class boundaries when the data remained mixed. Third, some activity and occupational effects were clearly stronger than others, suggesting selective, data-dependent interpretation rather than uniform emphasis across all variables.

These observations are important because they show that the framework is capable of producing both positive and negative findings, which is essential for interpretable and trustworthy AI in sleep medicine. Rather than generating only attractive narratives, the multi-agent pipeline retained non-significant, moderate, and overlapping patterns where appropriate.

### 3.5 Summary of main findings

Across both datasets, the proposed framework consistently identified three major findings. First, bedtime consistency, light exposure, caffeine intake, stress, heart rate, and body temperature were retained as clinically interpretable candidate determinants, but the updated pairwise bootstrap analyses showed weak or null-crossing associations with continuous sleep-duration and sleep-quality outcomes in the public dataset. Second, physical activity effects were not uniform: activity type influenced sleep disorder profiles, with agility drills, jump tests, and lateral moves more frequently aligning with insomnia, whereas endurance running showed a stronger association with sleep apnea. Third, high caffeine intake showed the clearest elevation in sleep-disorder risk, whereas stress, physiological strain, and occupational context jointly shaped broader disorder profiles with greater uncertainty. Additional covariate-adjusted analyses showed that the occupational heterogeneity observed in both the public dataset and the synthetic distributional robustness dataset remained directionally consistent after controlling for age, gender, and BMI category, although several occupation-specific estimates were attenuated and should be interpreted cautiously. These findings highlight the importance of multimodal integration of behavioral, physiological, demographic, and contextual determinants. The bootstrap confidence-interval and CV analyses further indicate that several candidate determinants had wide or null-crossing confidence intervals, supporting a more cautious and statistically transparent interpretation of the results. These bootstrap statistics are distinct from the separate LLM repeated-generation stability analysis. These findings should be interpreted as exploratory and hypothesis-generating rather than as evidence of causal mechanisms or clinical generalizability.

## 4 Discussion

This study addressed three clinically relevant questions in sleep medicine using an interpretable multi-agent LLM framework for multimodal analysis of heterogeneous determinants of sleep quality and sleep disorders. Specifically, we examined: (1) which lifestyle and behavioral determinants most strongly influence adult sleep quality and sleep duration; (2) how physical activity type and intensity relate to sleep disorder prevalence and risk; and (3) how caffeine intake, psychological stress, physiological factors, and occupational context jointly modulate sleep disorder profiles. These questions are difficult to address using conventional pipelines because standard statistical models are typically optimized for predefined, mostly linear relationships, whereas common machine learning models often improve prediction at the cost of interpretability. In contrast, the proposed framework was designed to support evidence-grounded analysis of interaction effects across heterogeneous domains while preserving transparency in the reasoning process.

For RQ1, the proposed framework retained bedtime consistency, caffeine intake, light exposure, stress, heart rate, and body temperature as clinically interpretable candidate determinants of sleep duration and sleep quality. However, the updated bootstrap results showed that their pairwise correlations with continuous sleep outcomes were weak and generally crossed the null value. Caffeine intake showed the most consistent directional trend among these behavioral variables, but its correlations with sleep duration and sleep quality remained small and non-significant. These results are still compatible with the literature summarized in Table 10, including evidence that irregular bedtimes are associated with shorter and poorer sleep, that caffeine can impair subsequent sleep through adenosine-related sleep-wake mechanisms, and that caffeine timing may be relevant to circadian/light-related effects (11, 39–42), but the present dataset does not by itself support strong standalone pairwise claims for these determinants. Taken together, the findings support a cautious behavioral–circadian interpretation in which these variables remain plausible domains for future study, while the current public dataset provides limited pairwise evidence for continuous sleep-duration or sleep-quality effects.

**Table 10:** External literature support for the principal findings of the proposed framework.

Key finding	Supporting result from prior studies	References
Bedtime consistency, light exposure, and caffeine intake were retained as plausible behavioral determinants, but the present pairwise data supported only weak or uncertain sleep-duration and sleep-quality associations	Irregular bedtimes are associated with shorter sleep duration and poorer subjective sleep quality, supporting continued evaluation of sleep timing even when the present dataset yields weak pairwise effects.	(11, 39)
	Caffeine can impair subsequent sleep, and adenosine-mediated sleep–wake regulation provides a physiological basis for caffeine-related arousal.	(41, 42)
	Caffeine and bright evening light can delay circadian timing.	(40)
The type of physical activity significantly influenced sleep disorder prevalence	High-intensity activity is associated with poorer sleep quality, consistent with greater insomnia risk than lower-intensity activity.	(43)
	Walking- or resistance-dominant activity patterns are associated with lower prevalence of obstructive sleep apnea symptoms than inactivity.	(44)
Caffeine intake, stress, and body temperature were retained as interacting risk domains, with high caffeine intake showing the clearest dataset-supported sleep-disorder risk signal	Higher caffeine-use scores correlate with poorer sleep quality and greater burnout among physicians, supporting caffeine–stress contributions to insomnia risk in workplace settings.	(45)
	Ambient temperature is associated with night-to-night obstructive sleep apnea severity, implicating thermoregulatory physiology in sleep-apnea burden.	(46)

For RQ2, the framework revealed modality-specific relationships between physical activity and sleep disorder profiles. Explosive or high-intensity activities, such as agility drills and jump tests, were more closely associated with insomnia-like patterns, whereas sustained endurance activity was more strongly linked to sleep apnea. This distinction is important because it suggests that physical activity should not be treated as a single homogeneous predictor in sleep medicine analysis. Instead, different activity modalities may place distinct demands on arousal regulation, recovery, and respiratory physiology. The identified pattern is consistent with external evidence summarized in Table 10, including reports that vigorous activity may be associated with poorer sleep quality and that walking- or resistance-dominant patterns may correspond to lower obstructive sleep apnea symptom prevalence than inactivity (43, 44). From a mechanistic perspective, the present findings support the interpretation that highly arousing or late-scheduled activities may exacerbate sleep-onset difficulties, whereas sustained endurance-related loading may interact differently with apnea burden. Importantly, the proposed multi-agent design did not simply report a generic association between physical activity and sleep; rather, it generated a more nuanced, interpretable account of activity-specific sleep disorder profiles.

For RQ3, the framework identified caffeine exposure, psychological stress, physiological measures, and occupational context as interacting domains for sleep-disorder profiling. High caffeine exposure produced the clearest elevation in overall sleep-disorder risk, whereas stress level and body temperature did not show reliable standalone risk-ratio effects after bootstrap uncertainty was considered. Occupational context also modulated disorder patterns, indicating that workplace routines and demands may influence how behavioral and physiological factors manifest as insomnia or sleep apnea. This interpretation is again supported by prior work summarized in Table 10, including reports that higher caffeine use among physicians co-occurs with poorer sleep and burnout, and that thermoregulatory conditions are associated with night-to-night variability in obstructive sleep apnea severity (45, 46). Notably, these are higher-order relationships spanning multiple domains; they are difficult to recover and explain using simple additive models alone. The present framework enabled coordinated analysis of such multivariate interactions while retaining explicit linkage to quantitative summaries and validation checks.

The additional covariate-adjusted analysis further refines the interpretation of the occupational findings. After controlling for age, gender, and BMI category, the occupation-related differences in sleep disorder subtype remained directionally consistent across both the public dataset and the synthetic distributional robustness dataset. This suggests that the occupational heterogeneity observed in Figures 4c, 5c was not solely driven by demographic or anthropometric composition. However, the adjusted analysis also showed that some occupation-specific estimates were attenuated and several confidence intervals overlapped, particularly in smaller occupational subgroups. Therefore, occupational context should be interpreted as an exploratory contextual correlate of sleep disorder subtype profiles rather than as an independent causal determinant. This more cautious interpretation strengthens the robustness of the finding while avoiding overstatement of occupation-specific effects.

An important contribution of this study is therefore methodological as well as empirical. The proposed large language model was not used as a free-form generator of plausible explanations, but as a coordinated set of agents with complementary roles: quantitative evidence extraction, physiology- and health-aware interpretation, and iterative validation against dataset-derived summaries. This structure improved interpretability, supported traceability between findings and underlying evidence, and reduced the likelihood that final claims were driven solely by prior model knowledge rather than the dataset itself. In this sense, the framework contributes to current efforts in the field to develop interpretable, trustworthy, and clinically meaningful AI systems for sleep medicine.

The findings also have broader relevance to the special issue theme. Although the present study does not directly model neurodegenerative disease trajectories or brain imaging biomarkers, it addresses a core challenge emphasized in this Research Topic: the need for novel multimodal AI and LLM-based frameworks capable of integrating heterogeneous data sources in sleep medicine. Ensemble deep learning frameworks for situational aspects-based annotation have shown that multi-source data fusion–combining behavioral signals with contextual metadata—enables robust classification even under pandemic-related data variability (47). This aligns with our framework's design for handling heterogeneous, multimodal sleep determinants. Because sleep disorders are increasingly linked to brain dysfunction, cognitive impairment, and neurological health more broadly, improved analytical characterization of sleep-related determinants may also support future work on the interface between sleep pathology and neurodegenerative processes. From this perspective, the current framework should be viewed as an interpretable and extensible methodological foundation for more comprehensive multimodal sleep medicine analysis.

It is important to emphasize that the present findings are associative rather than causal. The proposed framework identifies interpretable relationships, subgroup patterns, and interaction-related profiles across heterogeneous sleep-related variables, but it does not establish causal mechanisms, estimate counterfactual effects, or evaluate the efficacy of behavioral interventions. Therefore, variables such as bedtime consistency, caffeine intake, light exposure, stress, and physical activity type should be interpreted as associated factors or candidate domains for further clinical assessment, rather than as confirmed causal targets. Any claim that modifying these factors would reliably improve sleep outcomes would require prospective longitudinal evidence, randomized behavioral intervention studies, or formal causal inference designs. Accordingly, the translational value of the framework lies primarily in risk characterization, hypothesis generation, and evidence-grounded prioritization of variables for future clinical investigation.

Adult sleep health emerges from interacting behavioral, physiological, and occupational influences rather than isolated variables. By integrating these heterogeneous determinants into a transparent analytical workflow, the proposed framework provides a more comprehensive account of sleep quality and sleep disorder risk than conventional isolated-variable analysis. This positions the approach as both a methodological contribution to interpretable AI for sleep medicine and a practical analytical tool for data-driven understanding of clinically relevant sleep-related patterns.

### 4.1 Clinical decision-support scenario

To illustrate the potential clinical applicability of the proposed framework, we provide a decision-support scenario involving a hypothetical patient profile. Consider a software engineer presenting with an insomnia-dominant sleep complaint, high caffeine intake exceeding 250 mg/day, irregular bedtime timing, shortened sleep duration, and elevated perceived stress. The framework would not be used to diagnose the patient or prescribe treatment autonomously. Instead, it would generate an evidence-grounded prioritization summary to support clinician-led assessment.

In this scenario, the Data Analyst Agent would first identify the patient-relevant determinants most aligned with the dataset-derived patterns. Based on the present analyses, high caffeine intake would be highlighted as the clearest dataset-supported candidate risk marker, while low bedtime consistency, reduced light-exposure regularity, and elevated stress would be treated as plausible but more weakly supported contextual domains. The Physiology and Health Analyst Agent would then translate these patterns into a sleep-medicine interpretation: high caffeine intake may represent stimulant-related arousal, irregular bedtime may reflect circadian and behavioral dysregulation, and elevated stress may indicate hyperarousal that can maintain insomnia-like symptoms. The Validation Analyst Agent would check whether each proposed interpretation is supported by the extracted statistical evidence and would remove or qualify unsupported claims.

The resulting decision-support output would guide the clinician to prioritize intervention targets in a stepped and clinically cautious manner. First, the clinician would confirm the insomnia-dominant presentation and screen for symptoms suggesting obstructive sleep apnea or other medical sleep disorders when clinically indicated. Second, because high caffeine intake showed the clearest association with sleep-disorder risk in the present data, caffeine timing and total daily dose would be prioritized for assessment and modification. Third, irregular bedtime timing, light exposure, and stress-related arousal would be assessed as contextual contributors, for example through sleep diaries, morning light regularity, evening light hygiene, and stress-management strategies, while acknowledging that their pairwise associations in the current dataset were weak. Lower-priority variables, such as blood pressure, would not be emphasized solely on the basis of the present dataset unless patient-specific clinical findings suggested otherwise.

This vignette demonstrates that the proposed multi-agent framework is best understood as a clinician-facing prioritization aid. It converts multivariate analytical outputs into an interpretable list of candidate intervention domains while preserving the distinction between dataset-supported associations, physiologically plausible interpretations, and claims that remain unverified.

## 5 Conclusion

This study presented an interpretable multi-agent LLM framework for multimodal analysis of sleep quality and sleep disorders in sleep medicine. By integrating heterogeneous determinants spanning behavioral, physiological, physical-activity, and occupational domains, the proposed framework addressed an important limitation of conventional statistical and machine learning pipelines: their difficulty in simultaneously capturing higher-order interactions and preserving clinically meaningful interpretability. In contrast, the proposed system combined quantitative evidence extraction, domain-aware interpretation, and iterative validation within a unified analytical workflow.

Across the analyzed datasets, the framework consistently retained bedtime consistency, light exposure, caffeine intake, stress, heart rate, and body temperature as clinically interpretable candidate domains. However, the updated bootstrap analyses showed that several pairwise associations with continuous sleep-duration and sleep-quality outcomes were weak or null-crossing, indicating that these variables should be interpreted as exploratory and context-dependent rather than as strong standalone determinants. High caffeine intake showed the clearest dataset-supported association with elevated sleep-disorder risk, while physical activity type and occupational context contributed to broader disorder-profile patterns.

Beyond these empirical findings, the study contributes methodologically by showing that multi-agent LLMs can be used as an interpretable analytical framework for sleep medicine rather than only as generative tools. The coordinated use of a Data Analyst Agent, a Physiology and Health Analyst Agent, and a Validation Analyst Agent enabled transparent, evidence-linked reasoning over structured sleep-related data. This improves the traceability of claims, reduces unsupported interpretations, and strengthens the translational relevance of the resulting analyses.

Overall, the proposed framework provides a practical methodological pathway for multimodal AI-assisted sleep medicine research by supporting interpretable, evidence-grounded, and association-based analysis of heterogeneous sleep-related determinants. However, the framework should not be interpreted as providing causal inference or direct evidence for intervention efficacy. Its clinical utility at the current stage is best understood as supporting risk characterization, hypothesis generation, and identification of candidate modifiable domains for future prospective or experimental validation. Establishing whether behavioral changes in these domains can reliably improve sleep outcomes will require large-scale real-world clinical cohorts, longitudinal designs, randomized intervention studies, or formal causal inference approaches. Although the present study does not directly model neurodegenerative disease progression, the identified relationships are relevant to the broader clinical context in which sleep disorders, brain dysfunction, and cognitive impairment are increasingly recognized as interconnected. In this sense, the framework offers a useful foundation for future interpretable AI systems aimed at more comprehensive analysis of sleep disorders and their neurological consequences.

## 6 Limitations and future work

Several limitations should be acknowledged. First, although the proposed framework is designed for multimodal analysis, the present study integrates heterogeneous structured determinants rather than high-dimensional biomedical modalities such as polysomnography, neuroimaging, electrophysiology, genomics, or longitudinal electronic health records. Therefore, the current work should be interpreted as an interpretable multimodal determinant-integration framework for sleep medicine rather than a full biomarker-discovery system. Second, the study relied on a synthetic dataset inspired by real-world distributions rather than a large-scale clinical cohort. While this design enabled controlled evaluation of the analytical pipeline, further validation on real-world clinical and longitudinal data will be necessary to establish generalizability. Therefore, the current synthetic validation analysis should be interpreted as a controlled robustness and sensitivity analysis, not as evidence of external clinical generalizability.

Third, although the multi-agent architecture improves interpretability and evidence grounding, LLM-based systems remain dependent on the capabilities and biases of the underlying model. An additional limitation concerns LLM memorization and adversarial robustness. Although the framework constrains agent prompts to dataset-derived statistics and requires final claims to be traceable to structured evidence, these safeguards cannot fully eliminate the possibility that the underlying LLM may generate statements influenced by memorized prior knowledge or common sleep-medicine narratives. The Validation Analyst Agent reduces this risk by rejecting claims that cannot be mapped to numerical evidence, but it remains an LLM-based component and may itself be vulnerable to adversarially phrased inputs, misleading evidence summaries, or prompt-induced overgeneralization.

The present study did not conduct a comprehensive adversarial evaluation of the Validation Analyst Agent. Future work should therefore include adversarial stress tests in which the validation module is exposed to fabricated correlations, label-swapped disorder categories, unsupported causal claims, contradictory subgroup summaries, and deliberately misleading prompts. Such testing would help determine whether the validation layer can reliably reject unsupported interpretations under hostile or noisy analytical conditions. Future implementations may also combine LLM-based validation with rule-based evidence checkers or independent statistical scripts to further reduce memorization-driven or adversarially induced errors.

Fourth, the present analyses are associative rather than causal. Although the framework identifies interpretable relationships, interaction-related patterns, and subgroup profiles, it does not establish causal mechanisms, counterfactual effects, or behavioral intervention efficacy. The observed associations should therefore not be interpreted as evidence that modifying bedtime consistency, caffeine intake, light exposure, stress, or physical activity type would necessarily improve sleep outcomes. Instead, these variables should be viewed as candidate modifiable domains that may warrant further investigation. Finally, because the current study is primarily focused on sleep medicine, its neurodegenerative relevance is indirect rather than explicit. The results motivate future work on neurological consequences of sleep pathology, but they should not be interpreted as direct evidence for disease progression mechanisms in Alzheimer's disease, Parkinson's disease, or related disorders.

Future work should extend the framework toward richer clinical and translational settings. One important direction is the incorporation of real-world multimodal data streams such as actigraphy, wearables, polysomnography, neuroimaging, and electronic health records, which would allow more comprehensive modeling of sleep disorders and their neurological consequences. A second direction is longitudinal analysis, which could enable monitoring of sleep-related changes over time and support trajectory modeling for clinically relevant outcomes. A third direction is expansion of the agentic framework itself, for example by including additional agents focused on epidemiology, intervention design, mechanistic reasoning, or clinical decision support. Finally, future studies should evaluate the framework across more diverse demographic and occupational cohorts to improve robustness, fairness, and generalizability, and to move toward interpretable AI systems that are more directly usable in real-world sleep medicine practice.

## Data Availability

The original contributions presented in the study are included in the article/supplementary material, further inquiries can be directed to the corresponding author.
